# Wearable technology interventions in patients with chronic obstructive pulmonary disease: a systematic review and meta-analysis

**DOI:** 10.1038/s41746-023-00962-0

**Published:** 2023-11-27

**Authors:** Amar J. Shah, Malik A. Althobiani, Anita Saigal, Chibueze E. Ogbonnaya, John R. Hurst, Swapna Mandal

**Affiliations:** 1 Royal Free London NHS Foundation Trust, London, UK; 2 UCL Respiratory, University College London, London, UK; 3 King Abdulaziz University, Department of Respiratory Therapy, Faculty of Medical Rehabilitation Sciences, Jeddah, Makkah Saudi Arabia; 4 Institute of Child Health, University College London, London, UK

**Keywords:** Respiratory tract diseases, Disease prevention, Patient education

## Abstract

Chronic obstructive pulmonary disease (COPD) is the third leading cause of death and is associated with multiple medical and psychological comorbidities. Therefore, future strategies to improve COPD management and outcomes are needed for the betterment of patient care. Wearable technology interventions offer considerable promise in improving outcomes, but prior reviews fall short of assessing their role in the COPD population. In this systematic review and meta-analysis we searched ovid-MEDLINE, ovid-EMBASE, CINAHL, CENTRAL, and IEEE databases from inception to April 2023 to identify studies investigating wearable technology interventions in an adult COPD population with prespecified outcomes of interest including physical activity promotion, increasing exercise capacity, exacerbation detection, and quality-of-life. We identified 7396 studies, of which 37 were included in our review. Meta-analysis showed wearable technology interventions significantly increased: the mean daily step count (mean difference (MD) 850 (494–1205) steps/day) and the six-minute walk distance (MD 5.81 m (1.02–10.61 m). However, the impact was short-lived. Furthermore, wearable technology coupled with another facet (such as health coaching or pulmonary rehabilitation) had a greater impact that wearable technology alone. Wearable technology had little impact on quality-of-life measures and had mixed results for exacerbation avoidance and prediction. It is clear that wearable technology interventions may have the potential to form a core part of future COPD management plans, but further work is required to translate this into meaningful clinical benefit.

## Introduction

Chronic obstructive pulmonary disease (COPD) is the third leading cause of death worldwide and is characterised by poorly reversible airflow obstruction secondary to a significant exposure to noxious gases or particles, accompanied by respiratory symptoms^1,2^. Patients with COPD have an underlying chronic inflammatory state that contributes to multiple medical and psychological comorbidities. These comorbidities add to the individual burden of disease, contribute to frequent hospitalisations, and add to an ever-growing healthcare cost. Furthermore, the natural history of COPD is punctuated by exacerbations which accelerate lung decline, and lead to a decreased physical reserve, impaired quality of life and increased mortality^3,4^. Given the significant individual and global burden of COPD, there is an urgent need to find future strategies to improve COPD diagnosis, management, and outcomes to improve patient care and quality of life.

Wearable health technology can be defined as any electronic device that is worn close to or on the skin’s surface that detects and collects data with a means for retrieval. In recent years, the wearable health market has grown exponentially with an estimated market value of $29 billion in 2019, which is predicted to rise to nearly $ 200 billion by 2027^5,6^. Over the last two decades there have been several advancements in the use of wearables in the COPD population. In the main the focus has been on physical activity improvement by the use of activity trackers (pedometers and accelerometers). Wearables, such as continuous pulse oximetry devices have also been studied for their role in COPD monitoring. However, the reliability, accuracy and utility of the devices are still debated and few have made it into mainstream use^7^.

There have been several previous systematic reviews investigating the role of step-counters in promoting the mean daily step count in a COPD population. Both Qui et al.^8^ (*n* = 15 studies) and Armstrong et al.^9^ (*n* = 12 studies) found that step counter use increased physical activity compared to controls (standardised mean difference (SMD) = 0.57 (95%CI 0.31–0.84) and 0.53 (0.29–0.77) respectively). However, both reviews may be biased by including studies that did not mandate gold-standard spirometric diagnostic criteria for COPD and were limited by only including studies investigating step counters. Han et al. only focused on studies that lasted at least 12 weeks (*n* = 9) and showed a significant increase in physical activity of ≥793 steps/day^10^. Finally, Reilly et al.^11^ recently reviewed interventions to promote physical activity as assessed by step-count in chronic airways disease, but did not split the results by different disease groups. Only Quiet al.^8^ looked at physical capacity as assessed by the six-minute walk distance (6MWD) and no prior studies have investigated the role of wearables on other measures of physical activity or capacity such as time spent at various intensity levels and muscle strength. Moreover, no prior reviews have looked at whether wearable devices impact patient quality of life using standardised questionnaires.

In terms of other aspects of COPD management, a prior review by Al Rajeh and Hurst looked at whether monitoring physical parameters can predict COPD exacerbations. This review (*n* = 16) included a mix of wearable technology but only looked at intermittent rather than continuous monitoring. While the data was heterogenous, the authors concluded that monitoring physiological variables does have the potential to detect exacerbations^12^. Recent advances in this field have not yet been subject to systematic review.

To date, reviews have only focused on the role of wearables in physical activity improvement in the COPD population. However, the management of COPD includes other facets, such as smoking cessation, exacerbation prevention and quality of life improvement. It is still not clear whether wearable devices benefit COPD patients in all facets of their care. We therefore aimed to conduct a systematic review and meta-analysis, using gold standard diagnostic criteria for COPD, to assess the impact of wearable technology interventions on physical activity promotion, exercise capacity, exacerbation detection, smoking cessation, home self-management, disease progression, and quality of life.

## Results

### Literature search

**Fig. 1:**
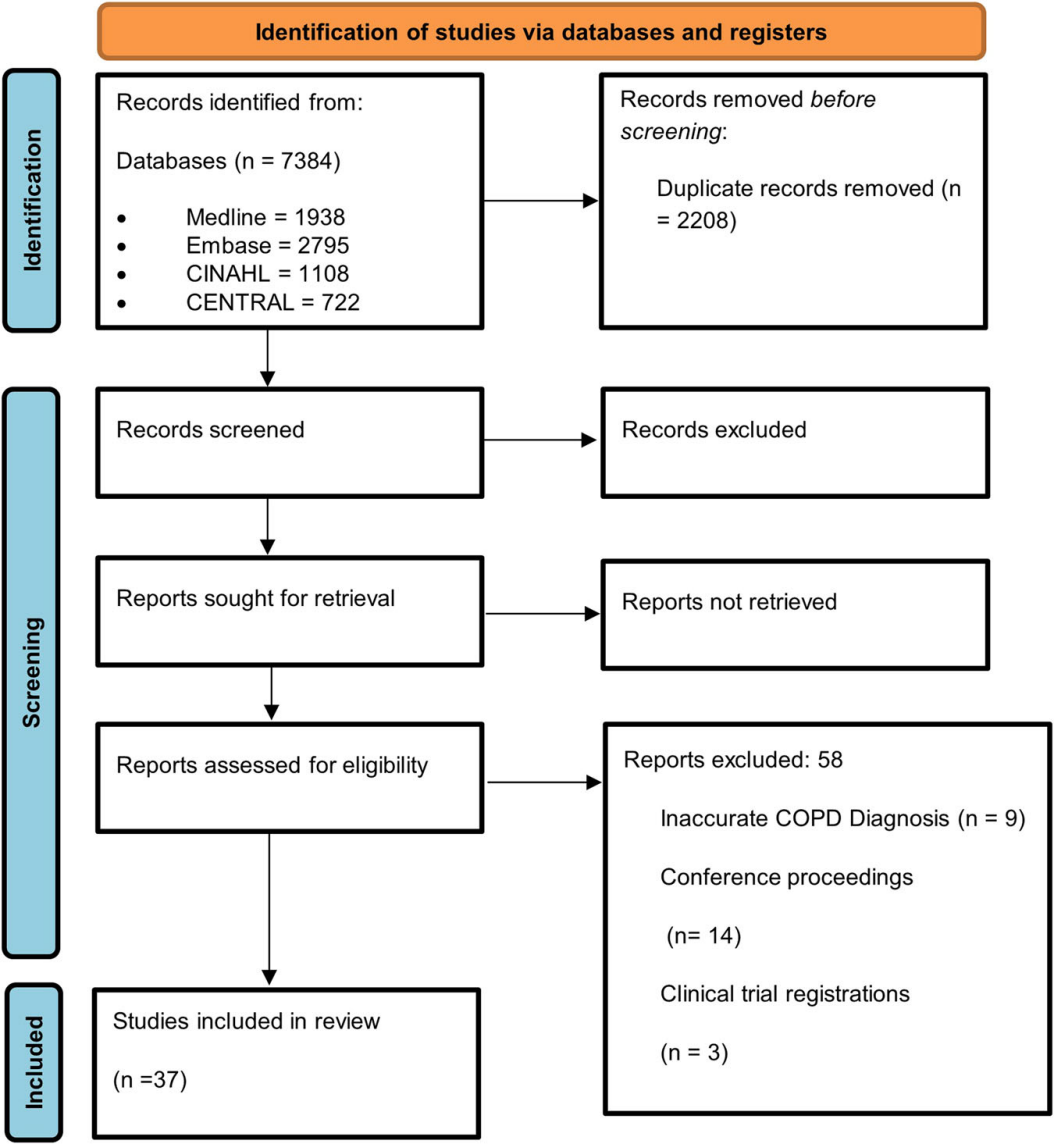
PRISMA flow chart for included studies.

The initial search generated 7396 studies. After the removal of duplicates and screening of titles and abstracts, 96 studies were sought for retrieval, but one study could not be accessed, and the author was not reachable. Therefore, 95 studies were assessed in full for eligibility according to the inclusion criteria. An additional 58 papers were excluded following full-text review and a total of 37 studies met all the inclusion criteria. Figure 1 shows the PRISMA flow chart and a full list of the excluded studies at full-text review, with reasons, which can be seen in Supplementary Methods.

A summary of the included studies is reported in Table 1. Thirty studies^13–42^ investigated the role of wearable technology (with or without other components) in improving physical activity outcomes (27 randomised controlled trials (RCTs)^14–18^^,20–28,30–33,35–42^ and three observational studies^19,29,34^). The studies included a total of 2955 patients, 69% male, with a median (IQR) sample size of 53 (32–143), mean (SD) age of 67 (6) years and a median (IQR) FEV1% predicted of 54 (45–59)%. For the RCTs the median (IQR) drop-out rate in the intervention group was 20% (10−29%), similar to 17% (10−28%) in the control group. Most RCTs used a per-protocol analysis (80%).

**Table 1:** Characteristics of the included studies.

Author, Year, Country	Study Design	Sampling/N/duration of intervention/COPD Severity	Intervention Group/wearable used/sample characteristics/Attrition rate	Control Group/sample characteristics/Attrition rate	Outcomes of interest	
					Outcome measure	Mean difference between groups (95%CI) from baseline to end of study duration Or Other difference measure
Al Rajeh et al.^44^	RCT Per Protocol	Stable COPD patients	Overnight pulse oximetry measurements (SpO2 and HR). Recorded every 4 seconds	Once daily measurement of HR and SpO2	Exacerbation detection using changes in HR and Saturations	Control group showed no statistically significant variation from baseline prior to an exacerbation. Intervention group showed significant variation from baseline for both heart rate and oxygen saturation pre-exacerbation.
		88 randomised 44 to intervention 44 to control	Nonin 3150 pulse oximeter			
		6 months or 1^st^ exacerbation	Exacerbated *N* = 13; Male = 7 (54%) Mean age ± SD = 71 ± 3	Exacerbated *N* = 14; Male = 4(29%) Mean age ± SD = 72 ± 3	Composite score (of changes in heart rate and oxygen saturations)	Composite score increased in control group for 1 day prior to exacerbation Composite score in intervention group increased for 7 days prior to exacerbation with a positive predictive value of 91.7%; sensitivity 84.6% and specificity of 81.8%
		FEV1% 52.9	Attrition rate: 52%	Attrition rate: 59%		
Al Rajeh et al.^a43^	OBS	Stable COPD patients	Overnight pulse oximetry measurements (SpO2 and HR). Recorded every 4 seconds. This is secondary analysis of Al Rajeh et al. (2020) described above	—	Oxygen saturation variability measures	Data presented as stable phase vs. exacerbation phase
					Mean SpO2 (mean (SD))	91.4 ± 1.89% vs. 90.6 ± 2.11%; *p* = 0.125
		13	Nonin 3150		Sample entropy	0.395 ± 0.101 vs. 0.505 ± 0.159; *p* = 0.029
		1^st^ exacerbation	*N* = 11; Male 7 (64%) Mean age ± SD = 72 ± 10		Detrended Fluctuation Aanlaysis (α1)	1.17 ± 0.110 vs. 1.15 ± 0.137; *p* = 0.555
		FEV1% 47.7	Attrition rate: 15%		Detrended Fluctuation Aanlaysis (α2)	1.04 ± 0.114 vs. 0.925 ± 0.107; *p* = 0.002
Altenburg et al.^14^	RCT Per Protocol	Stable COPD patients from both GP practices, secondary care and PR	12-week lifestyle physical activity counselling programme. Pedometer with feedback and motivation and 5 × 30 min counselling sessions for 3 months.	Usual Care	Median Daily Steps	Median (IQR) daily step change given: Intervention 218 (−1423 to 1863) Control −201 (−1809 to 1006)
					6-minute walk distance (m)	Median (IQR) at each time point given Intervention: Baseline: 454 (361 to 509) 15-months: 506 (422 to 571) Control: Baseline: 450 (351 to 530) 15-months: 468 (417 to 543)
		155 randomised 78 to intervention 77 to control	Digiwalker SW-2000, Yamax, Tokyo, Japan			
		15months	Demographics only given for whole groups: *N* = 155; Male 102 (66%) Median age (IQR) = 62 (54-69)	Demographics only given for whole groups: *N* = 155; Male 102 (66%) Median age (IQR) = 62 (54-69)		
		FEV1% 60	Attrition rate: 36%	Attrition rate: 34%		
Arbillaga-Etxarri et al.^15^	RCT Per Protocol	Stable COPD patients	‘Urban Training intervention’ – motivational interviewing, urban training walking trails, walking groups and a pedometer	Usual care – general health counselling and ELF information brochure (recommending ≥ 30 min moderate physical activity ≥ 5days/week	Mean daily steps	−136.00 (−768.20 to 496.20)
					Severe COPD Exacerbation (%)	Mean difference of 6% (control group 3% and intervention 9%)
		407 randomised 202 to intervention 205 to control	Onstep 50 Geonaute and Omron Pedometer			
					6-minute walk distance (m)	−3.00 (−17.13 to 11.13)
		12 months	Analysed *N* = 132; Male = 114 (86%) Mean age ± SD = 68 ± 9	Analysed *N* = 148; Male = 130 (88%) Mean age ± SD = 69 ± 8	COPD Assessment Test Score	0.00 (−1.08 to 1.08
					Hospital anxiety and depression scale -A	1 (0.41 to 1.59)
		FEV1% 57	Attrition rate: 35%	Attrition rate: 28%		
					Hospital anxiety and depression scale -D	−1 (−1.45 to −0.55)
Armstrong et al.^16^	RCT Per Protocol	Stable COPD patients during PR	Pedometer + motivational interview + individual daily step-count target + PR	Usual PR programme delivered as per British Thoracic Society	Clinical visit-PROactive physical activity in COPD instrument total score	8.00 (4.58 to 11.42)
					Mean daily steps	1016 (581 to 1451)
		60 randomised 31 to intervention 29 to control	Fitburg, Camden, London.			
					Movement intensity (VMU)	93.00 (44.09 to 141.91)
					Sedentary time (min)	−0.24 (−0.81 to 0.32)
		8 weeks	Analysed *N* = 24; Male 9 (37.5%) Mean age ± SD = 71 ± 9	Analysed *N* = 24; Male 9 (38%) Mean age ± SD = 73 ± 9		
					Light time (min)	22.00 (2.56 to 41.44)
		FEV1% 50.5	Attrition rate: 23%	Attrition rate: 17%		
					Mod-vigorous time (min)	0.42 (−0.16 to 0.99)
					6-minute walk distance (m)	16.00 (−8.12 to 40.12)
					Hand grip strength (Kg)	2.10 (0.62 to 3.58)
					Quadricep capacity (Kg)	0.63 (0.05 to 1.21)
					Sit-to-stand reps (number in 30 s)	1.00 (−0.34 to 2.34)
					COPD Assessment Test Score	−2.10 (−3.78 to −0.42)
Bently et al.^13^	RCT Per Protocol	Stable COPD patients during PR	SMART-COPD intervention consisted of an Android App and wearable activity tracking device with goal setting and feedback	Blinded activity tracker only	Mean daily step count	Lack of data to calculate difference
					Incremental Shuttle walk test	Lack of data to calculate difference
		30 randomised 19 to intervention 11 to control	Fitbit Activity device			
		8 weeks during PR and 8 weeks post	Analysed *N* = 10; Male 8/19 (42%) Median age (IQR) = 68 (63–72)	Analysed *N* = 6; Male 5/11 (45%) Median age (IQR) = 66 (60–70)		
		Not given	Attrition rate: 47%	Attrition rate: 45%		
Benzo et al.^17^	RCT Per Protocol	Stable COPD patients	Android tablet with health coaching using video guided exercises, measurement of daily steps and pulse oximetry during exercises	Usual care/Wait list for PR	Mean daily steps	631 (−143 to 1405)
					Sedentary time (min)	−29.90 (−84.70 to 24.90)
		154 randomised 78 to intervention 76 to usual care	Vivofit activity monitor (Garmin, Switzerland) Oximeter 3150 Wrist Ox2, Nonin Medical, Minnesota		Light intensity time (min)	21.00 (−24.50 to 66.50)
					Mod intensity time (min)	9.70 (−4.25 to 23.65)
					Self-management ability scale total score	4.10 (1.68 to 6.52)
		8 weeks	Start study *N* = 72; Male 34 (47%) Mean age ± SD = 69 ± 8	Study start *N* = 74; Male 37 (50%) Mean age ± SD = 69 ± 9		
		FEV1% 42.5	Attrition rate: 28%	Attrition rate: 17%		
Cooper et al.^45^	OBS	Stable COPD patients	Remote patient monitoring with daily saturations, spirometry, and symptom questionnaires. This was accompanied by an accelerometer worn all the time.	−	Exacerbation detection	Due to poor adherence – unable to calculate
			GeneActiv ® Accelerometer			
		17				
		12 months	*N* = 17; Male = 5 (29%) Mean age ± SD = 71 ± 7			
		FEV1% 56.8	Attrition rate: 53%			
Chen et al.^18^	RCT Per Protocl	Stable COPD patients	Pedometer with step count target	Weekly counselling where participants were encouraged to be active and walk ≥ 30 min/day	Mean daily step count	2358 (738 to 3978)
					6-minute walk distance (m)	−13.13 (−47.52 to 21.26)
		45 randomised 21 to intervention 24 to control	Pedometer (brand not mentioned)			
					COPD Assessment Test Score	−6.35 (−11.27 to −1.43)
					Modified Medical Research Council score	−0.11 (−0.89 to 0.66)
		6 weeks	Analysed *N* = 15; Male 13 (87%) Mean age ± SD = 74 ± 8	Analysed *N* = 11; Male 9 (82%) Mean age ± SD = 72 ± 11		
		FEV1% 52	Attrition rate: 29%	Attrition rate: 54%		
Cruz et al.^19^	OBS	Stable COPD patients	PR with exercise training, psychoeducation, and feedback on physical activity with a wearable monitor	−	Mean daily step count	220 (−565 to 1005)
					Mod-vigorous time (min)	−5.05 (−14.00 to 3.90)
			GT3X Activity monitor		Light intensity time (min)	−0.08 (−28.33 to 28.17)
		20			Sedentary time (min)	−9.6 (−38.06 to 18.86)
		3 months	Analysed *N* = 16; Male 11 (69%) Mean age ± SD = 66 ± 11		Standing time (min)	30.06 (5.27 to 54.85)
					Sitting time (min)	2.13 (−8.43 to 12.69)
		FEV1% 70.3	Attrition rate: 20%.			
De-Blok et al.^20^	RCT Per Protocol	Stable COPD patients referred to PR aged 40-80 years	Lifestyle physical activity counselling program with pedometer feedback and goal settings in addition to PR	Usual PR	Mean daily steps	567 (−663 to 1797)
					Chair stand test (n)	1.10 (−1.35 to 3.55)
					Arm curl test (n)	2.50 (−0.93 to 5.93)
		21 randomised 10 to intervention 11 to control	Yamax Digi-Walker SW-200 (Tokyo, Japan)		2-min step test (n)	15.00 (−0.99 to 30.99)
					St George’s Respiratory Questionnaire score	3.30 (−6.38 to 12.98)
		10 weeks	Randomised *N* = 10; Male 5 (50%) Mean age ± SD = 66 ± 10	Randomised *N* = 11; Male 4 (36%) Mean age ± SD = 63 ± 12		
		FEV1% 47.5	Attrition rate: 20%	Attrition rate: 27%		
Demeyer et al.^21^	RCT Per Protocol	Stable COPD and those who had had an exacerbation Not in PR	Tele-coaching with step counter, direct feedback and smartphone app giving activity goals and feedback	Standard leaflet explaining importance of physical activity with a 5–10-minute session explaining	Mean daily steps	1548 (1012 to 2084)
					Moderate time (min)	0.57 (0.35 to 0.80)
					Walking time (min)	17.00 (9.68 to 24.32)
		343 randomised 171 to intervention 172 to control	Fitbug Air		Movement intensity (m/s^2^)	0.09 (0.04 to 0.14)
		3 months	Analysed *N* = 159; Male 111/171 (65%) Mean age ± SD = 66 ± 8	Analysed *N* = 159; Male 108/172 (63%) Mean age ± SD = 67 ± 8	6-minute walk distance (m)	13.51 (3.55 to 23.47)
					Quadricep strength (Kg)	0.05 (−0.17 to 0.27)
		FEV1% 56	Attrition rate: 7%	Attrition rate: 8%	COPD Assessment Test Score	−0.47 (−1.89 to 0.95)
Geidl et al.^22^	RCT Intention-to-treat	COPD patients undergoing inpatient rehabilitation	Pedometer given during 3-weeks inpatient rehabilitation then continued after. Feedback and goal setting	3-weeks inpatient rehabilitation and patient education	Means daily steps	496 (−72 to 1063)
					Moderate time (min)	0.21 (−0.00 to 0.43)
					Sedentary time (min)	−0.02 (−0.23 to 0.20)
		327 randomised 167 to intervention 160 to control	Pedometer, brand not mentioned		St George’s Respiratory Questionnaire score	2.20 −1.12 to 5.52)
					COPD Assessment Test Score	−0.79 (−3.06 to 1.48)
		6 months	*N* = 167; Male = 115 (69%) Mean age ± SD = 58 ± 6	*N* = 160; Male = 110 (69%) Mean age ± SD = 58 ± 5		
		FEV1% 53.5	Attrition rate: ?%	Attrition rate: ?%		
Hawthorne et al.^46^	OBS	COPD patients post acute exacerbation admission	Equivital LifeMonitor to be worn on discharge for 6 weeks. This monitor continuously records respiratory rate, heart rate, skin temperature and physical activity every 15 seconds	−	Changes in the following measures 3 days prior to an exacerbation (*n* = 11)	
					Changes in heart rate	Increased by a mean 8.1 ± 0.7 beats per minute
		50 recruited	*N* = 31 Analysed; Male 16 (52%) Mean age ± SD = 69 ± 8			
					Changes in Respiratory rate	Increased by a mean 2.0 ± 0.2 breaths/min
		6 weeks	Attrition rate: 38%		Changes in skin temperature	Nil change
		FEV1%: 43.5				
					Changes in physical activity	Nil change
Hornikx et al.^23^	RCT Per Protocol	Severe COPD exacerbators post hospital discharge	Pedometer used post discharge to provide real-time feedback on step counts. Physical activity counselling telephone calls three times per week with new goals set based on step-count	Usual care (no rehabilitation or motivational messages). General advice about increased physical activity during inpatient stay	Mean daily steps	−29 (−969 to 911)
					Minutes walked	0.00 (−11.50 to 11.50)
					Movement intensity ((m/s^2^) / day)	−0.02 (−0.06 to 0.02)
					Quadricep strength (Kg)	0.28 (−0.48 to 1.05)
		30 randomised 15 to intervention 15 to control	Fitbit Ultra (San Francisco, California)		6-minute walk distance (m)	3.00 (−53.13 to 59.13)
					Modified medical research Council score (median and IQR)	Intervention: 0 (−1 to 0) Control: 0 (−1 to 0)
					COPD Assessment Test Score (median and IQR)	Intervention: −3 (−10 to 1) Control: −5 (−7 to 1)
		1 month	Overall demographic *N* = 15; Male = 8 (53%) Mean age ± SD = 66 ± 7 Note only 12 analysed	*N* = 15; Male 9 (60%) Mean age ± SD = 68 ± 6		
		FEV1% 43	Attrition rate: 20%	Attrition rate: 0%		
Hospes et al.^24^	RCT Per Protocol	Stable COPD patients (45–75 years)	Exercise counselling group: included motivational interviewing based on pedometer feedback	Usual care only	Mean daily steps	2152 (527 to 3777)
					Leg strength (?units)	1.90 (0.66 to 3.14)
		39 randomised 20 to intervention 19 to control	Pedometer (Digiwalker SW-2000, Yamax, Tokyo, Japan)		Arm strength (?units)	6.30 (4.58 to 8.02)
		12 weeks	Analysed *N* = 18; Male 10 (55%) Mean age ± SD = 63 ± 8	Analysed *N* = 17; Male 11 (65%) Mean age ± SD = 61 ± 9	Grip force (?units)	0.20 (−4.67 to 5.07)
					6-minute walk distance (m)	12.50 (−10.76 to 35.76)
		FEV1% 64.6	Attrition rate: 10%	Attrition rate: 11%		
					St George’s Respiratory Questionnaire score	−6.60 (−13.22 to 0.02)
Kato et al.^25^	RCT Per protocol	Stable COPD patients	Pedometer to record their number of steps and self-evaluate the cumulative daily step count. No target number given	Usual care with no diary or pedometer	Knee extension strength (WBI)	0.08 (−0.04 to 0.20)
		26 randomised 12 to intervention 14 to control	Omron HJ-205IT pedoemeter (Omron, Tokyo, Japan)		6-minute walk distance (m)	43.30 (−15.50 to 102.10)
					St George’s Respiratory Questionnaire score	−5.10 (−14.73 to 4.53)
		6months	Analysed 6; Male 5 (83%) Mean age ± SD = 74 ± 5	Analysed 5; Male 5 (100%) Mean age ± SD = 73 ± 5		
					COPD Assessment Test Score	−2.80 (−8.22 to 2.62)
		FEV1% Not given	Attrition rate: 50%	Attrition rate: 64%		
Kawagoshi et al.^26^	RCT Per Protocol	Stable COPD patients	Pulmonary rehabilitation programme with pedometer feedback and goal setting	Home based pulmonary rehabilitation program with 45 min monthly education programme	Time spent walking / day (min)	39.00 (0.72 to 77.28)
					Time spent standing / day (min)	11.70 (−16.83 to 40.23)
		39 randomised 19 to intervention 20 to control	Pedometer (Kens Liferecorder EX, Nagoya, Japan)			
					Time spent sitting/day (min	53.20 (−20.93 to 127.33)
		12 months	Analysed *N* = 12; Male 10 (83%) Mean age ± SD = 74 ± 8	Analysed *N* = 15; Male 14 (93%) Mean age ± SD = 75 ± 9	Time spent lying down / day (min)	−24.30 (−72.00 to 23.40)
		FEV1% 56.6	Attrition rate: 37%	Attrition rate: 25%	Quadricep strength (Kg)	2.90 (−3.42 to 9.22)
					BODE index	−1.76 (−6.25 to 2.73)
					6-minute walk distance (m)	−18.59 (−39.55 to 2.36)
					Medical research council score	−0.20 (−0.50 to 0.10)
Kohlbrenner et al.^27^	RCT Per Protocol	Stable COPD patients aged over 40, with FEV1 <50% predicted	Physical activity counselling and pedometer with feedback. Activity diary (step counts, daily activity and goal setting) with monthly calls for 3 months, then unsupported for further 9 months	Usual care with no diary and no pedometer	Mean daily steps	300 (−412 to 1012
					COPD Assessment Test Score	0.31 (−3.68 to 4.30)
					1 min sit to stand reps	1.50 (−2.02 to 5.02)
		74 randomised 37 to intervention 37 to control	Pedometer (Omron Healthcare Co. Kyoto, Japan)			
		12 months	Randomised *N* = 37; Male 27 (73%) Mean age ± SD = 67 ± 9	Randomised *N* = 37; Male 23 (62%) Mean age ± SD = 64 ± 9		
		FEV1% 35	Attrition rate: 22%	Attrition rate: 16%		
Mendoza et al.^28^	RCT Per protocol	Stable COPD patients	Pedometer with feedback and goal setting	General counselling monthly and advised to increased activity and walk 30 min/day. Paper diary	Mean daily steps	2942 (1881 to 4002)
					6-minute walk distance (m)	13.10 (1.24 to 24.96)
		102 randomised 52 to intervention 50 to control	Pedometer (PD724 Triaxial pedometer, Tanita, Tokyo, Japan)		St George’s Respiratory Questionnaire score	−5.00 (−9.60 to −0.40)
					COPD Assessment Test Score	−2.90 (−5.33 to −0.47)
		3 months	Randomised *N* = 52; Male 29 (56%) Mean age ± SD = 69 ± 10	Randomised *N* = 50; Male 33 (66%) Mean age ± SD = 68 ± 8		
					Modified medical research council score	0.20 (−0.12 to 0.52)
		FEV1% 66.1%	Attrition rate: 4%	Attrition rate: 6%		
Moy et al.^29^	OBS	Stable COPD patients	Every step counts walking program which included a pedometer giving feedback with goal setting and motivational messages	−	Mean daily steps	1263 (−268 to 2794)
					Modified medical research council score	−0.24 (−0.85 to 0.37)
		27	Pedometer – Omron HJ-720ITC			
		3 months	Recruited *N* = 27; Male 27 (100%) Mean age ± SD = 72 ± 8			
		FEV1% 55	Attrition rate: 11%			
Nguyen et al.^30^	RCT Intention to treat	Stable COPD patients completed PR	‘MOBILE-COAHED’ – collaborative monitoring of symptoms and exercise (via pedometer) and ongoing reinforcement feedback with weekly messages	‘MOBILE SELF-MONITORED’ – Symptom and exercise information (via pedometer) but no feedback and no reinforcement	Mean daily steps	−1626 (−3459 to 207)
					Incremental cycle test (watts)	−6.80 (−22.32 to 8.72)
					6-minute walk distance (feet)	−114.00 (−341.52 to 113.52)
		17 randomised 9 to intervention 8 to control	Omron HJ-112 digital pedometer (Omron Healthcare, Bannockburn, IL, USA)	Omron HJ-112 digital pedometer (Omron Healthcare, Bannockburn, IL, USA)		
					St George’s Respiratory Questionnaire score	8.90 (0.30 to 17.50)
		6 months FEV1% 40.55	Analysed *N* = 9; Male 3 (33%) Mean age ± SD = 72 ± 9	Analysed *N* = 8; Male 3 (38%) Mean age ± SD = 64 ± 12		
			Attrition rate: 0%	Attrition rate: 13%		
Nguyen et al.^47^	RCT Intention to Treat	COPD patients needing ED attendance	Physical activity coaching intervention – ‘Walk-on!’ Collaborative monitoring of physical activity step counts, semiautomated step goals and individualised reinforcement	Standard care with no contact with study team	Self-reported activity	−
					All cause acute care use and death	OR 1.05 (0.82 – 1.35)
		2707 randomised 1358 to intervention 1349 to control	Either Omron HJ329 pedometer or Tractivity accelerometer or Fitbit Alta		Hospitalisations	OR 0.84 (0.65 – 1.10)
					Observation stays	OR 0.92 (0.66 – 1.28)
		12 months	Randomised *N* = 1358; Male 642 (47%) Mean age ± SD = 72 ± 10	Randomised *N* = 1349; Male 610 (45%) Mean age ± SD = 72 ± 10	Emergency department visits	OR 1.07 (0.84 – 1.36)
					Death	OR 0.62 (0.35 – 1.11)
					COPD-related acute care use	OR 0.96 (0.68 – 1.35)
		FEV1% 61.2	Attrition rate: 76%	Attrition rate: 3%		
Nolan et al.^31^	RCT Per protocol	Stable COPD patients undergoing initial PR assessment	Pedometer plus PR, with individualised daily step-count target and weekly review	Standardised twice-weekly outpatient PR program	Mean daily step count	198 (−657 to 1054)
					Mod-intensity time (min)	−0.16 (−0.53 to 0.21)
		152 randomised 76 to intervention 76 to control	Pedometer – Yamax Digi-walker CW700; Yamax, Bridgnoth, UK		Shuttle walk distance (m)	20.00 (−28.91 to 68.91)
					Chronic resp Questionnaire	−7.00 (−22.92 to 8.92)
		6months	Randomised *N* = 76; Male 56 (74%) Mean age ± SD = 69 ± 9	Randomised *N* = 76; Male 54 (71%) Mean age ± SD = 68 ± 8		
		FEV1% 50.5	Attrition rate: 26%	Attrition rate: 25%		
Park et al.^32^	RCT Intention to treat	Stable COPD patients	Combination of group education sessions, prescribed individualised exercises for each participant, pedometer with step count record and symptom monitoring. Built-in smart phone application	Group education sessions and prescribed individual exercises	Mean daily steps	1189 (90 to 2287)
					6-minute walk distance (m)	15.41 (−20.01 to 50.83)
					Mod-intensity activity (%of time)	0.02 (0.01 to 0.03)
					Sedentary behaviour (% of time)	−0.04 (−0.07 to −0.01)
		44 randmoised 23 to intervention 21 to control	Pedometer brand not mentioned			
					Self-efficacy for managing chronic diseases score	−0.04 (−0.87 to 0.73)
					Exacerbation needing hospitalisation (%)	Intervention: 9.1% Control: 10%
		6 months	Analysed *N* = 22; Male 19 (86%) Mean age ± SD = 68 ± 10	Analsyed *N* = 20; Male 14 (70%) Mean age ± SD = 65 ± 11		
		FEV1% 65	Attrition rate: 4%	Attrition rate: 5%		
Robinson et al.^33^	RCT Intention to treat	Stable COPD patients	Pedometer with individualised step count goals + objective walking assessment and feedback + motivational messages + online community	Verbal encouragement to increase physical activity and an educational booklet	Mean daily step count	1312 (192 to 2432)
					6-minute walk distance (m)	−12.27 (−38.93 to 14.39)
		153 randomised 75 to intervention 78 to control	Fitbit Zip pedometer		St George’s Respiratory Questionnaire score	0.07 (−0.25 to 0.39)
					Modified medical research council score	−0.13 (−0.45 to 0.19)
		6 months	Randomised *N* = 75; Male 70 (93%) Mean age ± SD = 69 ± 7	Randomised *N* = 78; Male 72 (92%) Mean age ± SD = 70 ± 7		
					Acute exacerbation (%)	Intervention: 12% Control 9%
		FEV1%: 61%	Attrition rate: 20%	Attrition rate: 31%		
Spielmanns et al.^35^	RCT Intention to treat	Stable COPD patients post PR	Physical exercise training essions via the Kaia COPD App with an activity tracker. The purpose of he app was to individualise strength training and increase daily steps	Activity tracker but no access to COPD App	Median daily step count	Effect size 0.402 (IQR 0.131 to 0.617
					COPD assessment test score	−5.12 (−7.53 to −2.71)
					Sit-to-Stand repetitions	1.04 (−1.49 to 3.51)
		67 randomised 33 to intervention 34 to control	Activity tracker: Polar A370® Watch			
		6 months	Randomised *N* = 33; Male 17 (52%) Mean age ± SD = 66 ± 7	Randomised *N* = 34; Male 17 (50%) Mean age ± SD = 63 ± 8		
		FEV1% 44	Attrition rate: 9%	Attrition rate: 13%		
Sasaki et al.^34^	OBS	Stable COPD patients	Pedometer provided. For 8 weeks patients were asked to increase their step count as much as possible using the pedometer.	—	Mean daily step count	205 (−123 to 534)
		19	Pedometer: OMRON healthcare, Kyoto, Japan			
		8 weeks	Analysed *N* = 16; Male 13 (81%) Mean age ± SD = 73 ± 7			
		FEV1% 56	Attrition rate: 16%			
Valeiro et al.^36^	RCT Per Protocol	Following an acute exacerbation of COPD	Motivational interview with a personalised physical activity program with a pedometer and weekly telephone calls	Usual Care	Mean daily step count	2193 (595 to 3791)
					Sedentary time (hours)	−0.10 (−1.16 to 0.96)
		46 randomised 22 to intervention 24 to control	Pedometer brand not mentioned			
					Light-intensity time (min)	−16.00 (−32.73 tp 0.73)
					Mod-intensity time (min)	14.00 (−4.77 to 32.77)
		12 weeks	Analysed *N* = 20; Male 16 (80%) Mean age ± SD = 66 ± 10	Analysed *N* = 23; Male 16 (70%) Mean age ± SD = 66 ± 10		
					Quadricep strength (Kg)	1.00 (−2.27 to 4.27)
		FEV1% 46	Attrition rate: 10%	Attrition rate: 4%	6-minute walk distance (m)	29.00 (−16.36 to 74.36)
					COPD assessment test score	−3.00 (−5.77 to −0.23)
Varas et al.^37^	RCT Per protocol	Stable COPD patients with low physical activity level and no PR for 12 months	5-group sessions of physiotherapy + 8-week community program with exercise training + pedometer with daily step-target.Post intervention – asked to keep same step-count	5-group sessions of physiotherapy. Given a pedometer but no target or instructions	Mean daily step count	2547 (927 to 4167)
					Shuttle test time (min)	7.50 (4.32 to 10.68)
					Shuttle test distance (m)	624.40 (230.76 to 1018.04)
		40 randomised 21 to intervention 19 to control	OMRON walking style X Pocket HJ-320e, Omron Healthcare Inc, Illinois		St George’s Respiratory Questionnaire score	−5.50 (−8.20 to −2.80)
					Modified medical research council score	−0.30 (−0.65 to 0.05)
		12 months	Randomised *N* = 21; Male 18 (86%) Mean age ± SD = 70 ± 7	Randomised *N* = 19; Make 13 (68%) Mean age ± SD = 65 ± 9		
		FEV1% 49	Attrition rate: 19%	Attrition rate: 16%		
Vorrink et al.^38^	RCT Per protocol	Stable COPD patients	Patients wore a smartphone continuously on a belt which measured physical activity and set individual personalised goals set.	Usual Care	Mean daily step count	−77 (−763 to 609)
					Metabolic equivalent of task	0.05 (−0.10 to 0.20)
		183 randomised 102 to intervention 81 to control	Smartphone – HTC Desire A8181; HTC; Taoyuan, Taiwan			
					6-minute walk distance (m)	−3.20 (−14.51 to 8.11)
					BMI (kg.m^2^)	0.04 (−0.29 to 0.37)
		12months	Completed baseline investigations *N* = 84; Male 42 (50%) Mean age ± SD = 62 ± 9	Completed baseline investigation *N* = 73; Male 36 (49%) Mean age ± SD = 63 ± 8		
		FEV1% 56	Attrition rate: 39%	Attrition rate: 27%		
Wan et al.^39^	RCT Per protocol	Stable COPD patients	Pedometer and website where step counts uploaded weekly and individualised goal set with iterative step-count feedback and motivational content	Pedometer alone with no website and no step-count goals	Mean daily step count	804 (105 to 1503)
					6-min walk distance (m)	3.50 (−15.92 to 22.92)
					St George’s Respiratory Questionnaire score	−0.23 (−4.53 to 4.07)
					Modified medical research council score	−0.20 (−0.60 to 0.20)
		114 randomised 60 to intervention 54 to control	Omron HJ-720 ITC pedometer			
		3 months	Analysed *N* = 57; Male 56 (98%) Mean age ± SD = 68 ± 9	Analysed *N* = 52; Male 51 (98%) Mean age ± SD = 68 ± 8		
		FEV1% 62.6	Attrition rate: 5%	Attrition rate: 4%		
Wan et al.^a^^48^	RCT 2^o^ Analysis	Stable COPD patients 15 months (12 months post study completion)	Secondary analysis of Wan et al. 2017 dataset	Secondary analysis of Wan et al. 2017 dataset	Risk of acute exacerbations	Rate ratio 0.51 (0.31 to 0.85)
Widyastuti et al.^40^	RCT Per Protocol	Stable COPD	Fast-walking at least 30 minutes/day and pedometer for 6 weeks with goal setting and feedback	3x30min weekly sessions for 6 weeks of supervised exercise training on a treadmill. Encouraged to be more active at home with 30 min fast walking/day. No pedometer	Mean daily step count	264 (−823 to 1351)
					6-minute walk distance (m)	−20.80 (−48.89 to 7.29)
					COPD assessment tool score	1.20 (−0.51 to 2.91)
		40 randomised 20 to intervention 20 to control	Omron HJ 321, Omron Healthcare CoLtd, Kyoto, Japan			
		6 weeks	Analysed *N* = 18; Male 16 (89%) Mean age ± SD = 68 ± 7	Analysed *N* = 18; Male 15 (83%) Mean age ± SD = 69 ± 9		
		FEV1% exact value not given	Attrition rate: 10%	Attrition rate: 10%		
Wootton et al.^42^	RCT Intention to Treat	Stable COPD patients	Unsupervised maintenance walking exercise 3 days a week for 12 months. Telephone calls with biofeedback from a pedometer and progressive goal setting	Unsupervised maintenance walking exercise 3 days a week for 12 months	6-minute walk distance (m)	16.00 (−10.20 to 42.20)
					Endurance shuttle walk test time (s)	58.00 (−119.21 to 235.21)
					Incremental shuttle walk test distance (m)	−29.00 (−62.81 to 4.81)
		95 randomised 49 to intervention 46 to control	G-Sensor accelerometer, Pedometers Australia, Cannington, Australia			
					St George’s Respiratory Questionnaire score	−3.00 (−7.20 to 1.20)
		12 months	Randomised *N* = 49; Male 25 (51%) Mean age ± SD = 70 ± 7	Randomised *N* = 46; Male 30 (65%) Mean age ± SD = 69 ± 9		
		FEV1% 43	Attrition rate: 18%	Attrition rate: 24%		
Wootton et al.^41^	RCT Per protocol	Stable COPD patients	Unsupervised maintenance walking exercise 3 days a week for 12 months. Telephone calls with biofeedback via a pedometer and progressive goal setting.	Unsupervised maintenance walking exercise 3 days a week for 12 months	Mean daily step count	894 (74 to 1714)
					Total energy expenditure (kcal)	5.00 (−106.11 to 116.11)
		86 randomised 42 to intervention 44 to control	G-Sensor accelerometer, Pedometers Australia, Cannington, WA, Australia			
					Sedentary time (min)	4.00 (−30.60 to 38.60)
		12 months	Randomised *N* = 42; Male 30 (71%) Mean age ± SD = 70 ± 7	Randomised *N* = 44; Male 23 (52%) Mean age ± SD = 69 ± 9		
					Light intensity (min)	24.00 (−12.59 to 60.59)
		FEV1% 44	Attrition rate: 45%	Attrition rate: 55%	Moderate intensity (min)	−10.00 (−25.97 to 5.97)
					Vigorous intensity (min)	0.00 (−1.33 to 1.33)
Wu et al.^49^	OBS	Stable COPD patients67	Prediction system which was made of 4 components: 1. Wearable device (Fitbit Versa) 2. Home air quality sensing device (EDIMAX Airbox) 3. Lifestyle observation platform 4. Health application	—	7-day prediction system for early detection of COPD exacerbations	Accuracy 92.1% Sensitivity of 94% Specificity 90.4% AUROC > 0.9
		Exact value not given	*N* = 67; Male 59 (88%) Mean age ± SD = 67 ± 11			

^a^secondary analysis papers.

### Physical activity and exercise capacity

**Fig. 2:**
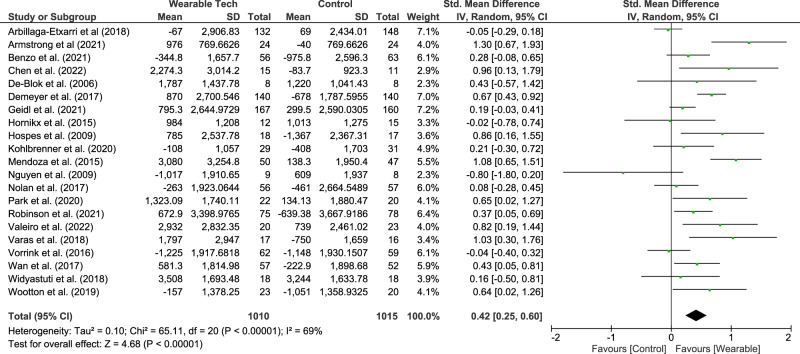
Meta-analysis results for mean daily step count reported.

The physical activity and exercise capacity metrics measured varied among studies (step counts, six-minute walk distance (6MWD), sedentary time, moderate-to-vigorous physical activity (MVPA) and quadricep strength). Meta-analysis showed that wearable technology interventions significantly increased the mean daily step count (21 studies^15–18,20–24,27,28,30–33,36–41^, 2025 participants, median (IQR) duration 3months (2.3−6 months)) with a standardised mean difference (SMD) of 0.42 (0.25–0.60), equating to a mean difference (95%CI) of 850 (494–1205) steps/day. This is illustrated in Fig. 2.

Multivariable meta-regression analysis with year of publication, participant age, baseline FEV1 (% predicted), type of pedometer used in the intervention and outcome measurement device (type of pedometer or accelerometer) explained 21% of the heterogeneity but was non-significant (residual *I*^2^ = 57%, *R*^2^ = 21%, *p* = 0.61). The full model results can be seen in Supplementary Table 1.

Subgroup analysis showed that studies where wearable technology was combined with additional health coaching (e.g., motivational interviewing or counselling) had a higher mean difference compared to studies where wearable technology was the only intervention (MD 998 (539–1456) steps/day vs. 243 (−341 – 801) steps/day). Moreover, studies that were of shorter duration (≤3 months) and those that used pedometers to measure their outcome variable had a higher overall mean difference. This is illustrated in Table 2.

**Table 2:** Meta-analysis results for mean daily step count and 6-min walk distance and CAT score based on different subgroups.

Subgroups	Mean daily step count			6-minute walk distance			CAT score		
	*N*	Effect size MD (95%CI)	I^2^ (%)	*N*	Effect size MD (95%CI)	I^2^ (%)	*N*	Effect size MD (95%CI)	I^2^ (%)
**Duration**									
≤3 months	11	1190 (715–1664)	67	9	10.13 (3.97–16.30)	6	5	−1.47 (−3.28 – 0.33)	74
>3 months	10	469 (34–905)	60	8	−0.80 (−8.43–6.82)	0	6	−1.82 (−3.74 – 0.11)	71
**Type of intervention**^a^									
Wearable technology^b^ with feedback±goal setting vs. usual care	2	243 (−314–801)	37	2	−1.54 (−12.64–9.56)	57	2	−1.09 (−3.19–1.01)	0
Wearable technology + health coaching^c^ vs. usual care	9	998 (539–1456)	55	7	11.75 (3.93–19.56)	0	2	−1.44 (−3.86–0.97)	61
Wearable technology + Pulmonary rehabilitation vs. Pulmonary rehabilitation alone	3	723 (191–1255)	33	2	15.66 (−7.04–38.36)	0	1	−2.10 (−3.78 – −0.42)	—
**Outcome measurement device**									
Pedometer	9	1582 (910–2255)	64						
Accelerometer	10	490 (114–866)	77						
**Severity of COPD**									
Moderate	12	1011 (539–1482)	78	11	5.97 (0.94–11.00)	17	6	−1.35 (−2.56 – 0.14)	59
Severe	8	649 (42–1255)	61	4	12·61 (−7.52–32.74)	0	3	−2·96 (−5·78–-0.14)	63

^a^This analysis excluded studies whereby the control arm was given a pedometer. Studies where the control arm had some counselling sessions or encouragement were excluded from this analysis. ^b^ all included studies used a step-counter as their intervention.

^c^Health coaching used to describe motivational interviewing±counselling±smart-phone access.

**Fig. 3:**
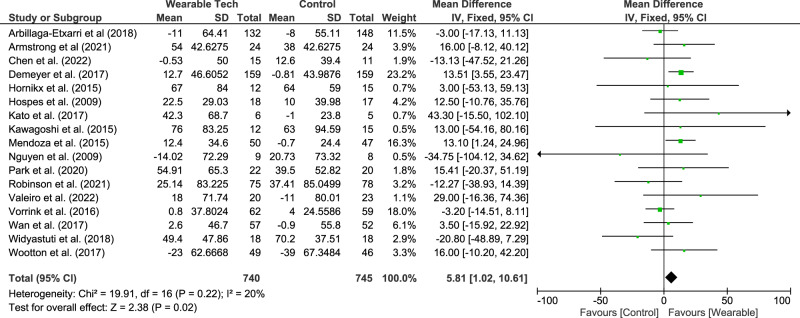
Meta-analysis results for the six-minute walk distance (m).

Meta-analysis showed that wearable technology interventions significantly increased the 6MWD (17 studies^15,16,18,21,23–26,28,30,32,33,36,38–40,42^, 1485 participants) with a mean difference (95%CI) of 5.81 m (1.02–10.61 m). This is illustrated in Fig. 3.

Subgroup analysis showed studies which were multi-component (wearable technology with health coaching) had a higher mean difference (11.75 m (3.93–19.56 m)). Studies of shorter duration (≤3 months) also had a higher mean difference compared to longer studies (10.13 m (3.97–16.30 m) vs. −0.80 m (−8.43 – 6.82 m)). (Table 2)

Wearable devices did not significantly impact sedentary time (4 studies^16,17,22,41^, 537 participants, SMD −0.07 (−0.24 −0.10)), MVPA time (7 studies^16,17,21,22,31,36,41^, 1010 participants, SMD 0.22 (−0.02–0.46)) and quadricep strength (5 studies^16,21,23,26,36^, 463 participants, SMD 0.15 (−0.03–0.33). The pooled effects of these can be seen in Supplementary Figs. 1 and 2. None of the observational studies^19,29,34^ found any difference in step count.

### Quality of life measures

**Fig. 4:**
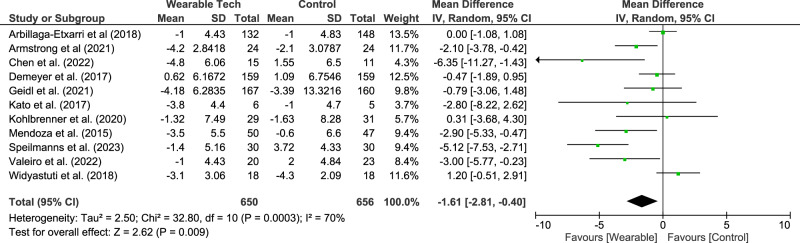
Meta-analysis results for the COPD Assessment Tool score.

Secondary outcome measures in 24 studies^15–18,20–33,35–37,39,40,42^ looked at changes in quality-of-life measures using validated questionnaires. The median duration (IQR) of these studies was 5.4 months (2.9–6 months). The primary outcome of all these studies was to determine the impact of wearables on physical activity. Meta-analysis showed that wearables were associated with a significant reduction in the COPD Assessment Tool (CAT) score (11 studies^15,16,18,21,22,25,27,28,35,36,40^, 1306 participants, median duration 3 months (2.31–6 months) by a mean difference (95%CI) of −0.99 (−1.59 to −0.40). This is illustrated in Fig. 4.

Subgroup analysis looking at study duration and type of intervention found no difference in the CAT score. (Table 2).

No significant differences were seen with the St George’s Respiratory Questionnaire (SGRQ) score (8 studies^20,24,25,30,33,37,39,42^, 469 participants, mean difference −1.73 (−4.90 to 1.44)); modified medical research council (mMRC) score (5 studies^18,28,33,37,39^, 418 participants, mean difference −0.10 (−0.30 to 0.11)). Two studies^15,16^ used the Clinical PROactive C-PPAC instrument that has previously been validated in COPD patients which requires both questionnaire and accelerometer data. Meta-analysis showed a significant improvement in the total score (mean difference 5.74 (1.85–9.62)). The pooled effects can be seen in Supplementary Figures 3-5.

### COPD self-management

Two studies investigated the role of wearables in COPD self-management through different scoring systems. Benzo et al.^17^, showed that the wearable intervention significantly increased the self-management ability scale (SMAS) with a mean difference of 4.10 (1.68–6.52); while Park et al.^32^, showed no significant difference when using the self-efficacy for managing chronic diseases (SEMCD) score with a mean difference of −0.04 (−0.87–0.73).

### COPD exacerbations

Ten studies^15,32,33,43–49^ investigated the role of wearable technology and COPD exacerbations. The studies included a total of 3660 patients, 69% male, a median (IQR) sample size of 78 (46–143), mean (SD) age of 69 (2) years and median (IQR) FEV1% predicted of 57 (53–61%). For the RCTs the median (IQR) drop-out rate in the intervention group was 36% (9–56%) and was 17% (4–30%) in the control group.

**Fig. 5:**
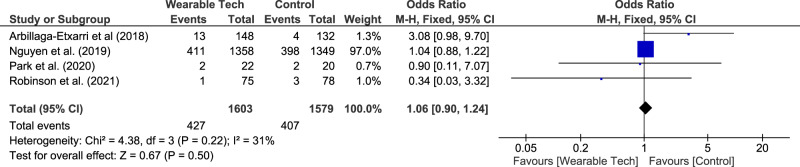
Meta-analysis investigating the association between wearable technology and COPD exacerbations.

Five RCTs^15,32,33,47,48^ assessed the association of pedometers and the rate of exacerbations needing hospitalisation. Meta-analysis of four studies^15,32,33,47^ (median follow-up duration 9 months) found no significant difference in the risk of hospitalisation from a COPD exacerbation (pooled OR 1.06 (0.90–1.24), *I*^2^ = 31%). This meta-analysis was dominated by one large study^47^ and is shown in Fig. 5. Wan et al.^48^ found pedometer use significantly reduced the risk of any acute COPD exacerbation over 15 months with a rate ratio of 0.51 (0.31–0.85).

It is worth noting that all four of these studies included multi-component interventions where wearable technology was combined with health coaching.

The remaining studies investigated the role of wearable technology in exacerbation prediction. Al Rajeh et al.^44^ found that a continuous oxygen saturation and heart rate composite score had a positive predictive value of 91.7% of exacerbation detection. Hawthorne et al.^46^ found significant changes in both heart rate and respiratory rate three days prior to an exacerbation but no changes detected in physical activity or skin temperature. Wu et al.^49^ conducted a telehealth study incorporating a wearable alongside a health application and home air quality device and algorithms combing these inputs could predict early detection of COPD exacerbations (sensitivity 94% and specificity 90.4%). Finally, Cooper et al.^45^ combined wearables with daily spirometry, however, due to a high attrition rate no data analysis was conducted.

### Study quality

The quality of the studies, as assessed by the Cochrane risk-of-bias tool^50^ can be seen in Supplementary Figure 6. Several studies had concerns in the domain looking at deviations from the intended interventions due to the per-protocol analysis employed, and the high drop-out rate in a large number of studies which would have affected the overall results. Studies had a low risk of bias in most of the other domains. The seven observational studies were of good quality and their Newcastle Ottawa Scale^51^ ratings can be seen in Supplementary Fig. 7.

## Discussion

This systematic review and meta-analysis, has shown: (1) wearable technology interventions significantly improved the mean daily step count in COPD patients over a median duration of 3 months, with an average effect size of 0.42, equating to a clinically important difference of 850 (494–1205) steps/day, [minimal important difference (MID) 600–1100 steps/day^52^]; (2) wearable technology significantly increased the 6MWD with a mean difference (95%CI) of 5.81 m (1.02–10.61 m), however, this was below the MID of 25 m^53^; (3) wearable technology significantly decreased the CAT score (mean difference −0.99 (−1.59 to −0.40)) but this did not reach the MID of −2 points^54^; (4) wearable technology may support COPD exacerbation detection, however, studies were heterogenous with mixed outcomes and had a high attrition rate, suggesting further work in this field is necessary to draw firm conclusions; (5) wearable technology had no significant impact on other activity or quality of life metrics.

To our knowledge, this is the largest and most up-to-date review investigating the effect of wearable technology interventions on physical activity and exercise capacity in a COPD population. The overall increase in mean daily step counts falls within the MID range and is higher than 600 steps/day, which has previously been shown to reduce the risk of hospitalisation in the COPD population^52^. Moreover, it is probable that wearable technology interventions have a larger positive impact on physical activity than exercise training programs, long-term oxygen therapy or neuromuscular stimulation^55,56^. It is worth noting that the studies were heterogenous (due to different intervention designs and wearable technology devices), and this could not be explained during multivariable meta-regression analysis, suggesting that findings need to be interpreted with caution. While our findings echo previous reviews^8–11^, key points of differences lies in our subgroup analyses: firstly, isolated pedometer use (with feedback and goal setting) has no significant difference to usual care (MD 243 (−314–801) steps/day) and prior reviews have not made this distinction; secondly, studies combining wearable technology with health coaching (e.g., motivational interviewing and counselling) had the largest mean difference of 998 steps/day (539–1456); thirdly, wearable technology in addition to pulmonary rehabilitation compared to pulmonary rehabilitation alone also had a significant improvement in mean daily step count (MD 723 (191–1255) steps/day). These results suggest, wearable technology interventions that include another facet (such as health coaching or pulmonary rehabilitation) are more likely to have a greater benefit to patients, then just giving patients a step-counter to use, even if goals are prescribed. Patients who have ongoing encouragement through telephone calls, counselling and motivational interviewing have a higher success rate and increased improvement.

**Fig. 6:**
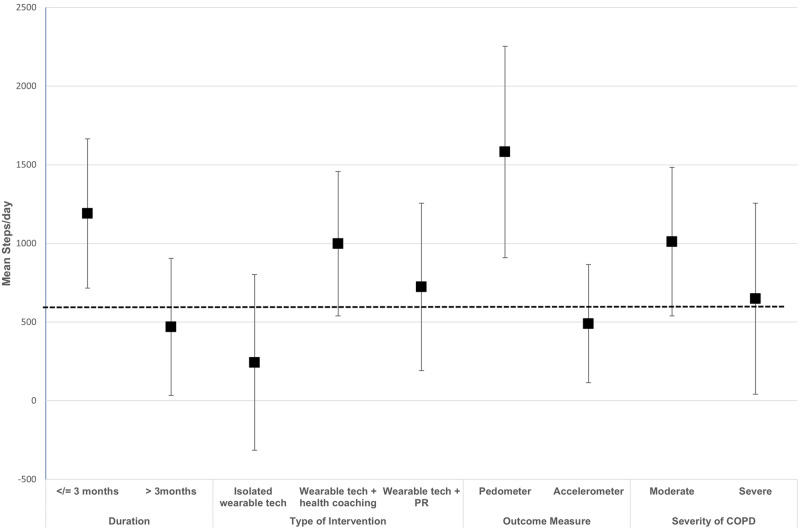
Subgroup analyses of the differences in mean daily steps achieved according to the minimum clinically important difference. Squares represent mean daily step count with error bars representing confidence interval. The dotted horizontal line represents the minimum clinically important difference (600 steps/day). The type of intervention is compared to usual care or pulmonary rehabilitation (PR).

Subgroup analysis also found that the increase in mean daily step count was lower in studies of greater than 3 months duration this increase in mean daily step count was lower in studies of more than 3months duration, in the severe COPD population and if an accelerometer was use for outcome measurement (Fig. 6). The latter may be explained by the fact that accelerometers are validated tools to measure step count in COPD patients, meaning pedometers may overestimate the true effect^57^.

While our study found no change in the time spent in MVPA, a recent international task force has suggested that the mean steps/day metric, irrespective of intensity, can be used as an overall surrogate for physical activity^58^. However, it should be noted that previous studies have also found that even a minimal increase in activity intensity (from very low to low), reduces the risk of COPD admissions and all-cause mortality^59^. Therefore, future wearable devices that incorporate and encourage changes in both overall activity and intensity are likely to be more beneficial in this population.

Our meta-analysis also showed an improvement in the 6MWD by 5.81 m (1.02–10.61 m), similar to previously published data by Qui et al.^8^,who found a change of 11.6 m. Both these values fall short of the MID of 25m^53^ but are higher than the change associated with telehealth interventions (1.3 m)^60^. Moreover, even a 6 m increase in 6MWD is associated with around a 4% risk reduction in all-cause and respiratory mortality in the COPD population^61^. Subgroup analysis showed that wearable technology combined with health coaching had a greater improvement with a mean difference of 11.75 m (3.93–19.56). This once again shows that multi-component interventions that include wearable technology are better that isolated devices.

This is the first review to our knowledge that has analysed the impact of wearables on quality-of-life measures in patients with COPD. Over a median duration of 3 months, wearables were associated with a significant reduction in CAT score by −0.99 points, below the MID of −2 points^54^ and thus unlikely to be clinically relevant, although it is worth noting a certain proportion of participants in these trials will have achieved the MID. While no study performed a responder analysis, a dedicated study investigating the association of wearable technology and CAT score may be useful. No improvement in any other quality-of-life measures were found. Similar findings were concluded from a recent umbrella review of five systematic reviews looking at the impact of activity trackers on psychosocial outcomes and quality of life in healthy participants and those with rheumatological and connective tissue disorders^62^. This may be because quality of life measures rarely consider participants’ perspectives or views of the actual activity. Two studies^15,16^ in this review incorporated the PROactive Physical Activity in COPD instrument (C-PPAC)^63^ which assesses patients’ experience of the amount of physical activity and the difficulty experienced with physical activity. Higher scores mean a better experience of the activity and less difficulty. Meta-analysis of these two studies showed wearable devices improved the difficulty score (i.e., patients had less difficulty with physical activity) and the total score. The difficulty dimension of the tool has a moderate-strong correlation with health status, chronic dyspnoea and exercise capacity^63^. These results need to be interpreted with some caution given only two recent studies have used this instrument. It is probable that quality of life is a key motivator for physical activity. Therefore, if wearables of the future can improve both quality of life while improving physical activity, it is more likely that patients will continue to use the devices and gain benefit in the longer term.

In this review five studies^15,32,33,47,48^ examined the association between use of physical activity monitors and the rate of exacerbations. A meta-analysis of four of these studies showed no significant difference with a pooled OR 1.06 (0.90–1.24), however, this should be interpreted with some caution as one study^47^ (*n* = 2707) was significantly larger than the others, and all studies used multi-component strategies, thus isolating the role of the wearable is difficult. It is also worth noting, that the primary aim of all the studies was to improve physical activity to decrease exacerbation risk, rather than using wearables to support detection of exacerbations.

Three studies^44,45,49^ used composite scores to predict exacerbation onset. Two of these studies^44,49^ showed high positive predictive values in exacerbation detection. While this is encouraging, some caution must be exercised. Al-Rajeh et al.^44^ had a high attrition rate and included only 13 patients in their final analysis, while Wu et al.^49^ incorporated a system combining environmental measures which can be quite costly and cumbersome to replicate in the non-research setting. However, it is probable that continuous monitoring of physiological parameters holds promise for exacerbation prediction, and future studies are needed to investigate wearables for this purpose.

Some limitations to our review should be noted. Firstly, the studies were heterogenous and used different objective outcomes and devices. This means that direct comparison between studies may be limited, however, the random effects model used in the meta-analysis and reporting standardised mean differences should reduce the bias attributed to this. Secondly, studies using pedometers differed in their approach to setting an individualised target step count. Thirdly wearables were often combined with other health interventions, such as motivational interviewing and walking programs, meaning the exact impact of the wearable device may be under or over-estimated. To account for this, we have performed a detailed subgroup analysis. Finally, many studies had a high drop-out rate which was not appropriately accounted for in the analysis. This led to attrition bias in most of the studies which will invariably impact the outcomes.

**Fig. 7:**
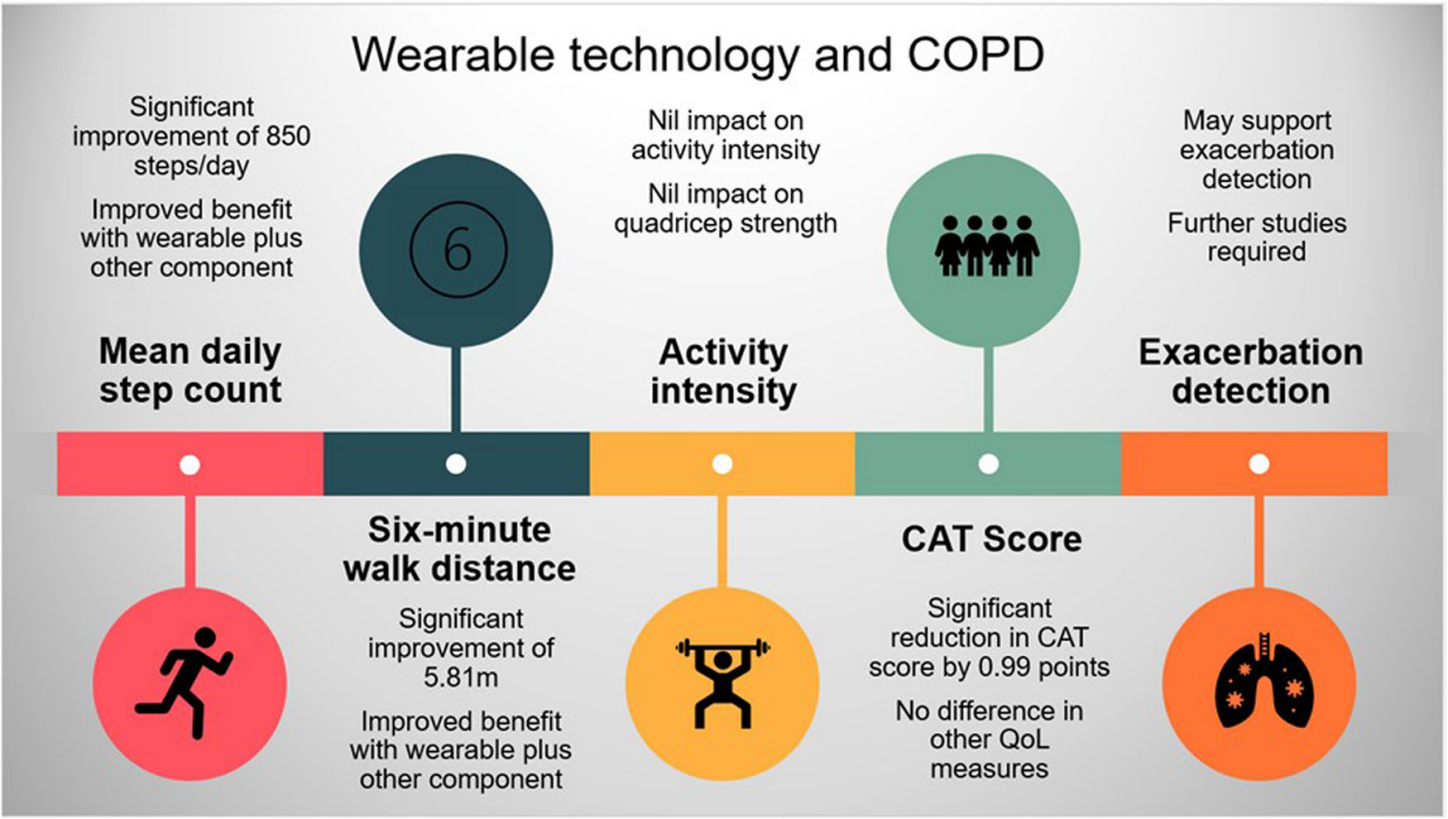
Summary infographic.

In conclusion, this systematic review and meta-analysis suggests that wearable device interventions significantly improve the mean daily step count and exercise capacity as measured by the 6MWD but does not impact activity intensity. The greatest benefit seems to be from multi-component interventions that include wearable technology and other facets, such as health coaching or pulmonary rehabilitation. Wearables have a limited impact on patient quality of life, and the gains seen in physical activity and exercise capacity are likely to be short-lived. Future work needs to focus on the positive reinforcement of wearable technology to simultaneously improve long term physical activity as well as quality of life measures. While the data is limited, wearables are likely to support the detection of COPD exacerbation, but further work in this field is required. The main findings from this review are highlighted Fig. 7. Overall, wearable technology has part of a multi-component intervention strategy seems to have the potential to become a core part of future COPD management and improve health outcomes, but further work is required for this to become a reality.

## Methods

### Search strategy and selection criteria

This systematic review and meta-analysis was conducted in accordance with the Preferred Reporting in Systematic Reviews and Meta-Analysis (PRISMA) guidelines and was prospectively registered on PROSPERO (registration number: CRD42022299706).

We included any article that investigated the use of wearable technology with or without other components in an adult COPD population with the following outcomes of interest: physical activity promotion, exercise capacity, exacerbation detection, smoking cessation, home self-management, disease progression and quality of life. The diagnosis of COPD had to be made with an adequate exposure history and post-bronchodilator spirometry showing either a forced expiratory volume in 1 s: forced vital capacity (FEV1:FVC) < 0.7 or <lower limit of normal (gold standard)^1^. Wearable technology was defined as any device that was worn/fitted to the subject’s body externally, which detected and collected data. The device needed a means to retrieve the data for analysis^64^. We excluded studies that were not in English, used other methods of COPD diagnosis, narrative reviews, non-research letters, abstracts, case reports, conference proceedings, theses, books, other systematic reviews (but searched the reference list), and studies looking at implantable or in-hospital wearables.

Following a scoping search in Google Scholar to identify relevant search terms, we did a systematic literature search of five database from inception to April 2023: MEDLINE (via OVID); EMBASE (via OVID); the Cumulative Index to the Nursing and Allied Literature (CINAHL, EBSCO host); Cochrane Central Register for Controlled Trials (CENTRAL); and the Institute of Electrical and Electronics Engineers (IEEE) Xplore digital library. We used an extensive search strategy under the supervision of an experienced health sciences librarian which included terms relating to COPD and wearable technology. Search strings used for MEDLINE (via OVID) can be seen in Supplementary Methods. We also conducted a full literature reference search of prior systematic reviews. The studies from the five databases were uploaded onto Endnote software and duplicates removed. Following this, the bibliographic data were loaded onto Rayyan^65^ for blind screening by two independent reviewers.

### Data analysis

Firstly, two authors (A.J.S., M.A.) independently screened titles and abstracts of studies against the inclusion criteria in a blinded fashion. Potentially eligible articles moved onto the next stage. Second, authors A.J.S. and M.A. independently assessed full texts of the potentially eligible articles for inclusion in the review. Third A.J.S. and M.A. developed a data extraction table including the year and country of publication, study settings, sample size and population, study duration patient demographics, intervention details, control group details, outcome data and attrition rates. A.J.S. and M.A. independently extracted data from each included article. Disagreements at each stage were resolved by discussion with S.M. The methodological quality of included studies was evaluated independently by A.J.S. and M.A. using the Cochrane risk of bias tool^50^ for randomised controlled trials and the Newcastle-Ottawa Scale (NOS) for observational studies^51^. Disagreements were resolved by SM. We attempted to contact study authors for unclear or missing information.

Physical activity and exercise capacity measurements were only included in the meta-analysis if they used an objective measurement tool (e.g., a pedometer/accelerometer). Subjective outcome measurements were not included in the meta-analysis.

Where meta-analysis was not possible due to significant heterogeneity, we undertook a narrative synthesis describing the included studies and their risk of bias.

Mean change scores with the corresponding standard deviation (SD) for the outcomes of interest were used in the meta-analysis to obtain the overall effect size, which was presented as either the mean difference or the standardised mean difference (SMD) with a 95% confidence interval. SMD was used where the same outcome of interest was measured by different devices. Where studies had not given the mean change scores, the mean change was calculated by subtracting the post-intervention mean from the baseline mean measure. The SD for changes from baseline was calculated using an imputed correlation coefficient of 0.80 with the following formula, derived from the Cochrane handbook (Eq. (1))^66^:.

1 $$S{D}_{{Change}}=\sqrt{S{D}_{{baseline}}^{2}+S{D}_{{Final}}^{2}-\left(2\times {Corr}\times S{D}_{{Baseline}}\times S{D}_{{Final}}\right)}$$

Heterogeneity was assessed by *I*^2^, with a value of ≥50% indicative of significant heterogeneity. If the data were heterogenous, a random-effects model was used rather than a fixed model. All statistical analysis was performed using the Cochrane Collaboration Review Manager software (version 5.4).

To understand the source of heterogeneity between studies, meta-regression analysis was performed on the mean daily step count pooled effect. Five covariates were included: age, publication year, FEV1% predicted, type of wearable used as part of the intervention, and the outcome measurement device. We conducted a mixed-effects meta-regression using Rstudio version 4.2.3. The regression analysis used a Knapp-Hartung modification and model fit was assessed by the Bayesian information criterion.

### Supplementary information

Supplementary Material Shah et al. (2023)

## Data Availability

A.J.S. and S.M. have full access to all of the data in the study and take responsibility for the data integrity and accuracy of the analysis. Data are Excevailable from the corresponding author upon reasonable request.
