# Supplementary material for: Wearable technology interventions in patients with chronic obstructive pulmonary disease: a systematic review and meta-analysis
Source: NPJ Digit Med. 2023 Nov 27;6:222. doi: 10.1038/s41746-023-00962-0 (PMC10682416; doi:10.1038/s41746-023-00962-0)
Supplement: Supplementary file 1 — Supplementary Material Shah et al. (2023) [file 41746_2023_962_MOESM1_ESM.pdf]

Home wearable technology in patients with chronic obstructive pulmonary disease: a systematic review and meta-analysis

Amar J Shah[1,2]; Malik A Athobiani[2,3]; Anita Saigal[1,2]; Chibueze E Ogbonnaya[4]; John R Hurst[1,2]; Swapna Mandal[1,2]

1. Royal Free London NHS Foundation Trust, London, UK

2. UCL Respiratory, University College London, UK

3. King Abdulaziz University, Department of Respiratory Therapy, Faculty of Medical Rehabilitation Sciences, Jeddah, Makkah, Saudi Arabia

4. Institute of Child Health, University College London, UK

Table of Contents

Supplementary Methods: Ovid MEDLINE Search Strategy 4

Supplementary Methods: Excluded studies and reason for exclusion. 8

Supplementary Figure 1: Meta-analysis results for moderate-vigorous activity intensity reported with the standardised mean differences. 13

Supplementary Figure 2: Meta-analysis results for quadricep strength reported with the standardised mean differences. 13

Supplementary Figure 3: Meta-analysis results for the St George's Respiratory Questionnaire (SGRQ) reported with the mean differences. 14

Supplementary Figure 4: Meta-analysis results for the modified medical research council (mMRC) score reported with the mean differences...14

Supplementary Figure 5: Meta-analysis results for the Clinical PROactive C-PPAC instrument score reported with the mean differences. 15

5a: Pooled results for the amount of exercise score 15

5b: Pooled results for the difficulty of exercise score 15

5c: Pooled results for total 15

Supplementary Figure 6: Cochrane-risk-of-bias tool for randomised controlled trials 16

Supplementary Figure 7: Newcastle Ottawa Scale ratings for the observational studies.......18

Supplementary Table 1: Multivariable meta-regression results for the mean daily step count 19

Supplementary Methods: Ovid MEDLINE Search Strategy

Search Strategy - Ovid MEDLINE(R) ALL <1946 to April 12, 2023>

#	Searches
1	exp Lung Diseases, Obstructive/
2	(chronic adj2 (air* adj2 obstruct*)).mp. [mp=title, abstract, original title, name of substance word, subject heading word, floating sub-heading word, keyword heading word, organism supplementary concept word, protocol supplementary concept word, rare disease supplementary concept word, unique identifier, synonyms]
3	((lung* or pulmon* or respirat* or bronchopulmon*) adj3 obstruct*).mp. [mp=title, abstract, original title, name of substance word, subject heading word, floating sub-heading word, keyword heading word, organism supplementary concept word, protocol supplementary concept word, rare disease supplementary concept word, unique identifier, synonyms]
4	(COAD or COBD or COPD).mp. [mp=title, abstract, original title, name of substance word, subject heading word, floating sub-heading word, keyword heading word, organism supplementary concept word, protocol supplementary concept word, rare disease supplementary concept word, unique identifier, synonyms]
5	((centriacinar* or centrilobular* or focal or panacinar* or panlobular* or pulmonar*) adj2 emphysem*).mp. [mp=title, abstract, original title, name of substance word, subject heading word, floating sub-heading word, keyword heading word, organism supplementary concept word, protocol supplementary concept word, rare disease supplementary concept word, unique identifier, synonyms]
6	exp Bronchitis/
7	bronchit*.mp. [mp=title, abstract, original title, name of substance word, subject heading word, floating sub-heading word, keyword heading word, organism supplementary concept word, protocol supplementary concept word, rare disease supplementary concept word, unique identifier, synonyms]
8	Exp Emphysema/
9	1 or 2 or 3 or 4 or 5 or 6 or 7 or 8
10	exp wearable electronic devices/ or exp fitness trackers/ or exp hearing aids/ or exp smart glasses/
11	((fit or fitness) adj3 tracker*).mp. [mp=title, abstract, original title, name of substance word, subject heading word, floating sub-heading word, keyword heading word, organism supplementary concept

	word, protocol supplementary concept word, rare disease supplementary concept word, unique identifier, synonyms]
12	fitbit.mp. [mp=title, abstract, original title, name of substance word, subject heading word, floating sub-heading word, keyword heading word, organism supplementary concept word, protocol supplementary concept word, rare disease supplementary concept word, unique identifier, synonyms]
13	((wear* or portabl* or home) adj3 activity*).mp. [mp=title, abstract, original title, name of substance word, subject heading word, floating sub-heading word, keyword heading word, organism supplementary concept word, protocol supplementary concept word, rare disease supplementary concept word, unique identifier, synonyms]
14	(activity* adj3 monitor*).mp. [mp=title, abstract, original title, name of substance word, subject heading word, floating sub-heading word, keyword heading word, organism supplementary concept word, protocol supplementary concept word, rare disease supplementary concept word, unique identifier, synonyms]
15	pedometer*.mp. [mp=title, abstract, original title, name of substance word, subject heading word, floating sub-heading word, keyword heading word, organism supplementary concept word, protocol supplementary concept word, rare disease supplementary concept word, unique identifier, synonyms]
16	((apple or smart) adj3 watch*).mp. [mp=title, abstract, original title, name of substance word, subject heading word, floating sub-heading word, keyword heading word, organism supplementary concept word, protocol supplementary concept word, rare disease supplementary concept word, unique identifier, synonyms]
17	((apple* or smart*) adj3 (telephone* or mobile* or cell*)).mp. [mp=title, abstract, original title, name of substance word, subject heading word, floating sub-heading word, keyword heading word, organism supplementary concept word, protocol supplementary concept word, rare disease supplementary concept word, unique identifier, synonyms]
18	exp Biosensing Techniques/
19	10 and 18
20	(wear* adj3 (ECG or electrocardiogram)).mp. [mp=title, abstract, original title, name of substance word, subject heading word, floating sub-heading word, keyword heading word, organism supplementary concept word, protocol supplementary concept word, rare disease supplementary concept word, unique identifier, synonyms]

21	(wear* adj3 ("blood pressure*" or hyperten*)).mp. [mp=title, abstract, original title, name of substance word, subject heading word, floating sub-heading word, keyword heading word, organism supplementary concept word, protocol supplementary concept word, rare disease supplementary concept word, unique identifier, synonyms]
22	(acceleromet*).mp. [mp=title, abstract, original title, name of substance word, subject heading word, floating sub-heading word, keyword heading word, organism supplementary concept word, protocol supplementary concept word, rare disease supplementary concept word, unique identifier, synonyms]
23	((wear* or portabl* or home) adj10 (biosens* or sensor* or track*)).mp. [mp=title, abstract, original title, name of substance word, subject heading word, floating sub-heading word, keyword heading word, organism supplementary concept word, protocol supplementary concept word, rare disease supplementary concept word, unique identifier, synonyms]
24	(Wear* adj3 monitor*).mp. [mp=title, abstract, original title, name of substance word, subject heading word, floating sub-heading word, keyword heading word, organism supplementary concept word, protocol supplementary concept word, rare disease supplementary concept word, unique identifier, synonyms]
25	((wear* or portabl* or home*) adj3 technolog*).mp. [mp=title, abstract, original title, name of substance word, subject heading word, floating sub-heading word, keyword heading word, organism supplementary concept word, protocol supplementary concept word, rare disease supplementary concept word, unique identifier, synonyms]
26	((wear* or portabl* or home*) adj3 (garment* or cloth* or shirt* or t?shirt* or blouse* or vest* or underwear)).mp. [mp=title, abstract, original title, name of substance word, subject heading word, floating sub-heading word, keyword heading word, organism supplementary concept word, protocol supplementary concept word, rare disease supplementary concept word, unique identifier, synonyms]
27	exp Textiles/
28	exp oximetry/ or exp blood gas monitoring, transcutaneous/
29	oximetr*.mp. [mp=title, abstract, original title, name of substance word, subject heading word, floating sub-heading word, keyword heading word, organism supplementary concept word, protocol supplementary concept word, rare disease supplementary concept word, unique identifier, synonyms]
30	((wear* or portabl* or home*) adj3 patch*).mp. [mp=title, abstract, original title, name of substance word, subject heading word, floating sub-heading word, keyword heading word, organism

	supplementary concept word, protocol supplementary concept word, rare disease supplementary concept word, unique identifier, synonyms]
31	10 or 11 or 12 or 13 or 14 or 15 or 16 or 17 or 19 or 20 or 21 or 22 or 23 or 24 or 25 or 26 or 27 or 28 or 29 or 30
32	9 and 31
33	Limit 32 to (English language)

Supplementary Methods: Excluded studies and reason for exclusion.

Inaccurate COPD diagnosis

Bender BG, Depew A, Emmett A, et al. A Patient-Centered Walking Program for COPD. *Chronic Obstr Pulm Dis* 2016; **3**(4): 769-77.

Lin WY, Verma VK, Lee MY, Lin HC, Lai CS. Prediction of 30-Day Readmission for COPD Patients Using Accelerometer-Based Activity Monitoring. *Sensors (Basel)* 2019; **20**(1).

Martinez CH, Moy ML, Nguyen HQ, et al. Taking Healthy Steps: rationale, design and baseline characteristics of a randomized trial of a pedometer-based Internet-mediated walking program in veterans with chronic obstructive pulmonary disease. *BMC Pulm Med* 2014; **14**: 12.

Moy ML, Collins RJ, Martinez CH, et al. An Internet-Mediated Pedometer-Based Program Improves Health-Related Quality-of-Life Domains and Daily Step Counts in COPD: A Randomized Controlled Trial. *Chest* 2015; **148**(1): 128-37.

Moy ML, Martinez CH, Kadri R, et al. Long-Term Effects of an Internet-Mediated Pedometer-Based Walking Program for Chronic Obstructive Pulmonary Disease: Randomized Controlled Trial. *J Med Internet Res* 2016; **18**(8): e215.

Orme MW, Weedon AE, Saukko PM, et al. Findings of the Chronic Obstructive Pulmonary Disease-Sitting and Exacerbations Trial (COPD-SEAT) in Reducing Sedentary Time Using Wearable and Mobile Technologies With Educational Support: Randomized Controlled Feasibility Trial. *JMIR Mhealth Uhealth* 2018; **6**(4): e84.

Riis HC, Jensen MH, Cichosz SL, Hejlesen OK. Prediction of exacerbation onset in chronic obstructive pulmonary disease patients. *J Med Eng Technol* 2016; **40**(1): 1-7.

Tabak M, Vollenbroek-Hutten MM, van der Valk PD, van der Palen J, Hermens HJ. A telerehabilitation intervention for patients with Chronic Obstructive Pulmonary Disease: a randomized controlled pilot trial. *Clin Rehabil* 2014; **28**(6): 582-91.

Wu R, Liaqat D, de Lara E, et al. Feasibility of Using a Smartwatch to Intensively Monitor Patients With Chronic Obstructive Pulmonary Disease: Prospective Cohort Study. *JMIR Mhealth Uhealth* 2018; **6**(6): e10046.

Conference Proceedings

PEDOMETER AS A TOOL FOR QUALITY OF LIFE IMPROVEMENT IN COPD PATIENTS. *Respirology* 2018; **23**(S2): 244-5.

Armstrong M, Hume E, McNeillie L, et al. P241 A pilot RCT assessing the inclusion of physical activity counselling to standard care pulmonary rehabilitation in patients with COPD. *Thorax* 2021; **76**(Suppl 1): A219-A.

Cao D, Zhang Z, Liang H, et al. Application of a Wearable Physiological Monitoring System in Pulmonary Respiratory Rehabilitation Research. 2018 11th International Congress on Image and Signal Processing, BioMedical Engineering and Informatics (CISP-BMEI); 2018 13-15 Oct. 2018; 2018. p. 1-6.

Collins R, Martinez CH, Kadri R, et al. An Internet-Mediated, Pedometer-Based Walking Program Improves HRQL In Veterans With COPD. B96 HIGHLIGHTS IN PULMONARY REHABILITATION: 2013: A3642-A.

Demeyer H, Waschki B, Polkey M, et al. The survival effect of physical activity in patients with COPD: every step counts. *European Respiratory Journal* 2017; **50**(suppl 61): OA512.

Etxarri AA, Gimeno-Santos E, Balcells E, et al. Effectiveness of an intervention of Urban Training in patients with COPD: a randomized controlled trial. *European Respiratory Journal* 2017; **50**(suppl 61): OA513.

Goldstein RL, Rivera PNC, Kadri R, Cooper JAD, Richardson CR, Moy ML. Results from a Multi-Site Web-Based Physical Activity Intervention in COPD: Between Group and Site Differences. A94 EXPANDING OUR HORIZONS; LEADING RESEARCH IN PULMONARY REHABILITATION: 2020: A2507-A.

Kantorowski A, Kadri R, Richardson CR, Gagnon D, Garshick E, Moy M. Internet-Mediated, Pedometer-Based Physical Activity Intervention Reduces Risk of Future Acute Exacerbations in COPD: A Randomized Trial. C17 PULMONARY REHABILITATION 2019: A4274-A.

Kantorowski A, Teylan M, Kadri R, et al. Patterns of Change in Daily Step Count Among COPD Patients Enrolled in a 3-Month Physical Activity Intervention. C17 ADVANCES IN PHYSICAL ACTIVITY, PULMONARY REHABILITATION, AND EXERCISE TRAINING: A4939-A.

Maddocks M, Canavan JL, Jones SE, et al. Pedometer-Directed Step Count Targets as an Adjunct to Pulmonary Rehabilitation in COPD: A Randomized Controlled Trial. B109 HIGHLIGHTS AND ADVANCES IN PULMONARY REHABILITATION: A7862-A.

Martinez CH, Kadri R, Roman P, et al. Long-Term Effects of An Internet-Mediated Pedometer-Based Walking Program in COPD: A Randomized Controlled Trial. B16 NEW RANDOMISED CONTROLLED TRIALS IN PULMONARY REHABILITATION: A2457-A.

Mendoza L, Aguilera M, Espinoza J, et al. Effects of program of physical activity enhancement using pedometers in COPD patients. *European Respiratory Journal* 2013; **42**(Suppl 57): P1837.

Mongiardo MA, Finer EB, Rivera PNC, Goldstein RL, Moy ML. Baseline Functional Status Is Associated with Response to a Web-Based Physical Activity Intervention in COPD. C14 C014 NEW INSIGHTS IN PULMONARY REHABILITATION: A1160-A.

Tiwari A, Liaqat S, Liaqat D, Gabel M, de Lara E, Falk TH. Remote COPD Severity and Exacerbation Detection Using Heart Rate and Activity Data Measured from a Wearable Device. *Annu Int Conf IEEE Eng Med Biol Soc* 2021; **2021**: 7450-4.

Clinical Trial Registrations

Bernocchi P, Scalvini S, Galli T, et al. A multidisciplinary telehealth program in patients with combined chronic obstructive pulmonary disease and chronic heart failure: study protocol for a randomized controlled trial. *Trials* 2016; **17**(1): 462.

Evans CN, Volpp KG, Polsky D, et al. Prediction using a randomized evaluation of data collection integrated through connected technologies (PREDICT): Design and rationale of a randomized trial of patients discharged from the hospital to home. *Contemp Clin Trials* 2019; **83**: 53-6.

Orme M, Weedon A, Esliger D, et al. Study protocol for Chronic Obstructive Pulmonary Disease-Sitting and ExacerbAtions Trial (COPD-SEAT): a randomised controlled feasibility trial of a home-based self-monitoring sedentary behaviour intervention. *BMJ Open* 2016; **6**(10): e013014.

Study did not use a wearable device / Wearable not part of the intervention

Ballal T, Heneghan C, Zaffaroni A, et al. A pilot study of the nocturnal respiration rates in COPD patients in the home environment using a non-contact biomotion sensor. *Physiol Meas* 2014; **35**(12): 2513-27.

Cheng SWM, Alison J, Stamatakis E, et al. Six-week behaviour change intervention to reduce sedentary behaviour in people with chronic obstructive pulmonary disease: a randomised controlled trial. *Thorax* 2022; **77**(3): 231-8.

Effing T, Zielhuis G, Kerstjens H, van der Valk P, van der Palen J. Community based physiotherapeutic exercise in COPD self-management: a randomised controlled trial. *Respir Med* 2011; **105**(3): 418-26.

Fernandez-Granero MA, Sanchez-Morillo D, Leon-Jimenez A. Computerised Analysis of Telemonitored Respiratory Sounds for Predicting Acute Exacerbations of COPD. *Sensors (Basel)* 2015; **15**(10): 26978-96.

Simmich J, Mandrusiak A, Smith ST, Hartley N, Russell TG. A Co-Designed Active Video Game for Physical Activity Promotion in People With Chronic Obstructive Pulmonary Disease: Pilot Trial. *JMIR Serious Games* 2021; **9**(1): e23069.

Does not meet outcome of interest.

Bowler R, Allinder M, Jacobson S, et al. Real-world use of rescue inhaler sensors, electronic symptom questionnaires and physical activity monitors in COPD. *BMJ Open Respir Res* 2019; **6**(1): e000350.

Buekers J, Theunis J, De Boever P, et al. Wearable Finger Pulse Oximetry for Continuous Oxygen Saturation Measurements During Daily Home Routines of Patients With Chronic Obstructive Pulmonary Disease (COPD) Over One Week: Observational Study. *JMIR Mhealth Uhealth* 2019; **7**(6): e12866.

Chawla H, Bulathsinghala C, Tejada JP, Wakefield D, ZuWallack R. Physical activity as a predictor of thirty-day hospital readmission after a discharge for a clinical exacerbation of chronic obstructive pulmonary disease. *Ann Am Thorac Soc* 2014; **11**(8): 1203-9.

Colantonio S, Govoni L, Dellacà RL, Martinelli M, Salvetti O, Vitacca M. Decision Making Concepts for the Remote, Personalized Evaluation of COPD Patients' Health Status. *Methods Inf Med* 2015; **54**(3): 240-7.

Crook S, Büsching G, Keusch S, et al. The association between daily exacerbation symptoms and physical activity in patients with chronic obstructive pulmonary disease. *Int J Chron Obstruct Pulmon Dis* 2018; **13**: 2199-206.

Davies HJ, Bachtiger P, Williams I, Molyneaux PL, Peters NS, Mandic DP. Wearable In-Ear PPG: Detailed Respiratory Variations Enable Classification of COPD. *IEEE Trans Biomed Eng* 2022; **69**(7): 2390-400.

Dias A, Gorzelniak L, Schultz K, et al. Classification of exacerbation episodes in chronic obstructive pulmonary disease patients. *Methods Inf Med* 2014; **53**(2): 108-14.

Ehsan M, Khan R, Wakefield D, et al. A longitudinal study evaluating the effect of exacerbations on physical activity in patients with chronic obstructive pulmonary disease. *Ann Am Thorac Soc* 2013; **10**(6): 559-64.

Emokpae LE, Emokpae RN, Jr., Bowry E, et al. A wearable multi-modal acoustic system for breathing analysis. *J Acoust Soc Am* 2022; **151**(2): 1033.

Faria I, Gaspar C, Zamith M, et al. TELEMOLD project: oximetry and exercise telemonitoring to improve long-term oxygen therapy. *Telemed J E Health* 2014; **20**(7): 626-32.

Hataji O, Kobayashi T, Gabazza EC. Smart watch for monitoring physical activity in patients with chronic obstructive pulmonary disease. *Respir Investig* 2016; **54**(4): 294-5.

Holland AE, Mahal A, Hill CJ, et al. Home-based rehabilitation for COPD using minimal resources: a randomised, controlled equivalence trial. *Thorax* 2017; **72**(1): 57-65.

Hurst JR, Donaldson GC, Quint JK, Goldring JJ, Patel AR, Wedzicha JA. Domiciliary pulse-oximetry at exacerbation of chronic obstructive pulmonary disease: prospective pilot study. *BMC Pulm Med* 2010; **10**: 52.

Kantorowski A, Wan ES, Homsy D, Kadri R, Richardson CR, Moy ML. Determinants and outcomes of change in physical activity in COPD. *ERJ Open Res* 2018; **4**(3).

Kuhn M, Kohlbrenner D, Sievi NA, Clarenbach CF. Increasing Daily Physical Activity and Its Effects on QTc Time in Severe to Very Severe COPD: A Secondary Analysis of a Randomised Controlled Trial. *Copd* 2022; **19**(1): 339-44.

Levy J, Álvarez D, Del Campo F, Behar JA. Machine learning for nocturnal diagnosis of chronic obstructive pulmonary disease using digital oximetry biomarkers. *Physiol Meas* 2021; **42**(5).

Moore R, Berlowitz D, Denehy L, Jackson B, McDonald CF. Comparison of pedometer and activity diary for measurement of physical activity in chronic obstructive pulmonary disease. *J Cardiopulm Rehabil Prev* 2009; **29**(1): 57-61.

Moy ML, Teylan M, Weston NA, Gagnon DR, Garshick E. Daily step count predicts acute exacerbations in a US cohort with COPD. *PLoS One* 2013; **8**(4): e60400.

Ney JP, Robinson SA, Richardson CR, Moy ML. Can Technology-Based Physical Activity Programs for Chronic Obstructive Pulmonary Disease Be Cost-Effective? *Telemed J E Health* 2021; **27**(11): 1288-92.

Rice KL, Schmidt MF, Buan JS, Lebahn F, Schwarzock TK. AccuO2 oximetry-driven oxygen-conserving device versus fixed-dose oxygen devices in stable COPD patients. *Respir Care* 2011; **56**(12): 1901-5.

Robinson SA, Shimada SL, Quigley KS, Moy ML. A web-based physical activity intervention benefits persons with low self-efficacy in COPD: results from a randomized controlled trial. *J Behav Med* 2019; **42**(6): 1082-90.

Robinson SA, Wan ES, Shimada SL, Richardson CR, Moy ML. Age and Attitudes Towards an Internet-Mediated, Pedometer-Based Physical Activity Intervention for Chronic Obstructive Pulmonary Disease: Secondary Analysis. *JMIR Aging* 2020; **3**(2): e19527.

Rubio N, Parker RA, Drost EM, et al. Home monitoring of breathing rate in people with chronic obstructive pulmonary disease: observational study of feasibility, acceptability, and change after exacerbation. *Int J Chron Obstruct Pulmon Dis* 2017; **12**: 1221-31.

Shah SA, Velardo C, Farmer A, Tarassenko L. Exacerbations in Chronic Obstructive Pulmonary Disease: Identification and Prediction Using a Digital Health System. *J Med Internet Res* 2017; **19**(3): e69.

Shany T, Hession M, Pryce D, et al. A small-scale randomised controlled trial of home telemonitoring in patients with severe chronic obstructive pulmonary disease. *J Telemed Telecare* 2017; **23**(7): 650-6.

Zhu Z, Barnette RK, Fussell KM, Michael Rodriguez R, Canonico A, Light RW. Continuous oxygen monitoring--a better way to prescribe long-term oxygen therapy. *Respir Med* 2005; **99**(11): 1386-92.

Nil reply from author for further information

Verwey R, van der Weegen S, Spreeuwenberg M, Tange H, van der Weijden T, de Witte L. A pilot study of a tool to stimulate physical activity in patients with COPD or type 2 diabetes in primary care. *J Telemed Telecare* 2014; **20**(1): 29-34.

Supplementary Figure 1: Meta-analysis results for moderate-vigorous activity intensity reported with the standardised mean differences.

Study or Subgroup	Wearable Tech Mean	Wearable Tech SD	Wearable Tech Total	Control Mean	Control SD	Control Total	Weight	Std. Mean Difference IV, Random, 95% CI
Armstrong et al (2021)	3	7.1046	24	0	7.1046	24	10.0%	0.42 [-0.16, 0.99]
Benzo et al. (2021)	-7.7	33.2	56	-17.4	44.2	63	15.6%	0.24 [-0.12, 0.61]
Demeyer et al. (2017)	8	19.1528	159	-3	19.1528	159	20.1%	0.57 [0.35, 0.80]
Geidl et al. (2021)	4.9	15.98	167	1.5	15.76	160	20.3%	0.21 [-0.00, 0.43]
Nolan et al. (2017)	2	52.2775	56	12	71.6074	57	15.3%	-0.16 [-0.53, 0.21]
Valeiro et al. (2022)	28	29.46	20	14	33.33	23	9.3%	0.43 [-0.17, 1.04]
Wootton et al. (2019)	-11	27.75	23	-1	25.6402	20	9.4%	-0.37 [-0.97, 0.24]
**Total (95% CI)**			**505**			**506**	**100.0%**	**0.22 [-0.02, 0.46]**

Heterogeneity: Tau² = 0.06; Chi² = 17.21, df = 6 (P = 0.009); I² = 65%
Test for overall effect: Z = 1.83 (P = 0.07)

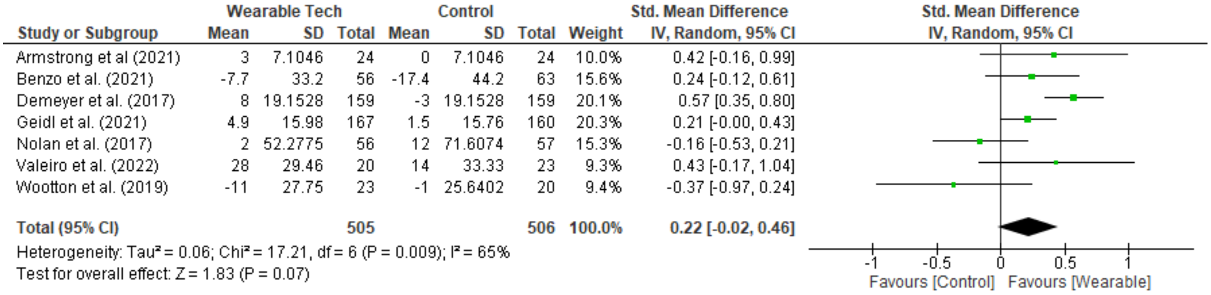

Supplementary Figure 2: Meta-analysis results for quadricep strength reported with the standardised mean differences.

Study or Subgroup	Wearable Tech Mean	Wearable Tech SD	Wearable Tech Total	Control Mean	Control SD	Control Total	Weight	Std. Mean Difference IV, Fixed, 95% CI
Armstrong et al (2021)	5	3.7891	24	2.5	4.0259	24	9.9%	0.63 [0.05, 1.21]
Demeyer et al. (2017)	0.102	9.27	159	-0.356	7.8782	159	69.3%	0.05 [-0.17, 0.27]
Hornikx et al. (2015)	0.54	1.22	12	0.04	2.03	15	5.7%	0.28 [-0.48, 1.05]
Kawagoshi et al. (2015)	5.1	8.57	12	2.2	8	15	5.7%	0.34 [-0.42, 1.11]
Valeiro et al. (2022)	2	4.84	20	1	6.08	23	9.3%	0.18 [-0.42, 0.78]
**Total (95% CI)**			**227**			**236**	**100.0%**	**0.15 [-0.03, 0.33]**

Heterogeneity: Chi² = 3.72, df = 4 (P = 0.45); I² = 0%
Test for overall effect: Z = 1.62 (P = 0.10)

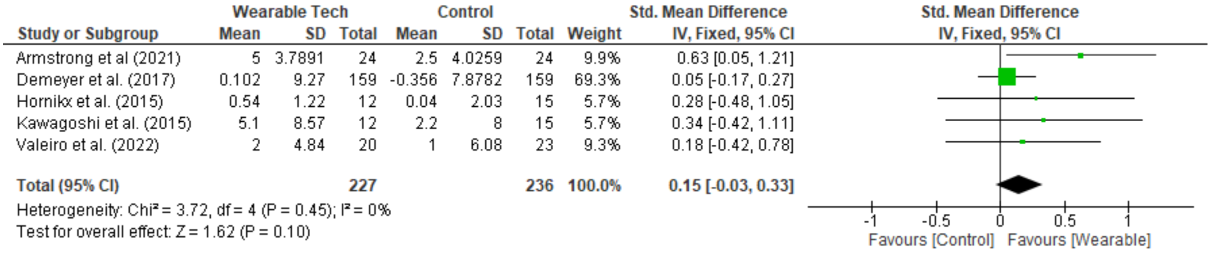

Supplementary Figure 3: Meta-analysis results for the St George's Respiratory Questionnaire (SGRQ) reported with the mean differences.

Study or Subgroup	Experimental Mean	Experimental SD	Experimental Total	Control Mean	Control SD	Control Total	Weight	Mean Difference IV, Random, 95% CI
De-Blok et al. (2006)	-2.8	8.157	8	-6.1	11.3436	8	7.5%	3.30 [-6.38, 12.98]
Hospes et al. (2009)	-3.5	8.26	18	3.1	11.37	17	12.0%	-6.60 [-13.22, 0.02]
Kato et al. (2017)	-6.6	9.7	6	-1.5	6.5	5	7.6%	-5.10 [-14.73, 4.53]
Nguyen et al. (2009)	3.4	8.92	9	-5.5	9.14	8	8.8%	8.90 [0.29, 17.51]
Robinson et al. (2021)	-13.05	31.0903	75	-15.13	28.4383	78	7.8%	2.08 [-7.37, 11.53]
Varas et al. (2018)	-2.3	3.8	17	3.2	4.1	16	21.4%	-5.50 [-8.20, -2.80]
Wan et al. (2017)	-0.9	9.9	57	-0.67	12.7	52	17.3%	-0.23 [-4.53, 4.07]
Wootton et al. (2017)	1	9.3804	49	4	11.3533	46	17.5%	-3.00 [-7.20, 1.20]
**Total (95% CI)**			**239**			**230**	**100.0%**	**-1.73 [-4.90, 1.44]**

Heterogeneity: Tau² = 10.29; Chi² = 16.28, df = 7 (P = 0.02); I² = 57%
Test for overall effect: Z = 1.07 (P = 0.28)

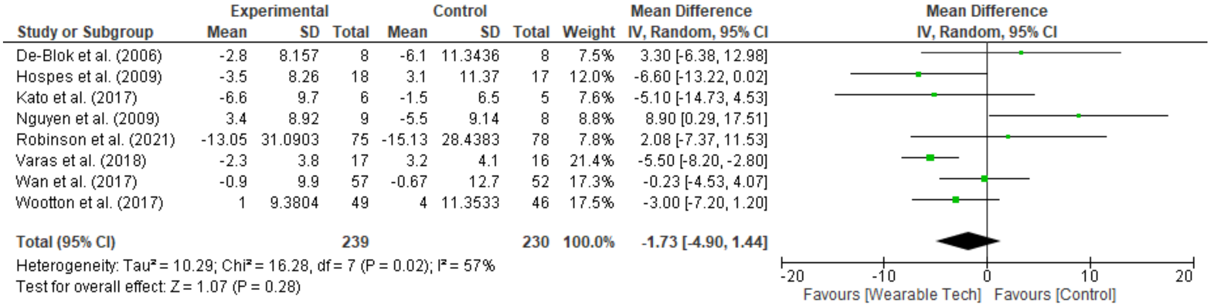

Supplementary Figure 4: Meta-analysis results for the modified medical research council (mMRC) score reported with the mean differences.

Study or Subgroup	Wearable Tech Mean	Wearable Tech SD	Wearable Tech Total	Control Mean	Control SD	Control Total	Weight	Mean Difference IV, Fixed, 95% CI
Chen et al. (2022)	0.13	0.74	15	0.21	0.58	11	12.5%	-0.08 [-0.59, 0.43]
Hornikx et al. (2015)	0	0	15	0	0	15		Not estimable
Mendoza et al. (2015)	0.2	0.7	50	0	0.9	47	31.1%	0.20 [-0.12, 0.52]
Robinson et al. (2021)	-0.17	1.8187	75	0.07	1.8547	78	9.5%	-0.24 [-0.82, 0.34]
Varas et al. (2018)	-0.2	0.4	17	0.1	0.6	16	26.3%	-0.30 [-0.65, 0.05]
Wan et al. (2017)	0.1	1	57	0.3	1.1	52	20.6%	-0.20 [-0.60, 0.20]
**Total (95% CI)**			**214**			**204**	**100.0%**	**-0.09 [-0.27, 0.09]**

Heterogeneity: Chi² = 5.05, df = 4 (P = 0.28); I² = 21%
Test for overall effect: Z = 0.99 (P = 0.32)

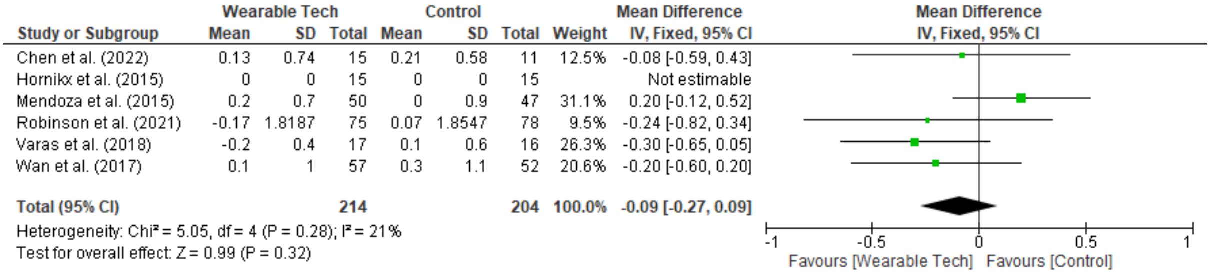

Supplementary Figure 5: Meta-analysis results for the Clinical PROactive C-PPAC instrument score reported with the mean differences.

5a: Pooled results for the amount of exercise score

	Experimental			Control				Mean Difference
Study or Subgroup	Mean	SD	Total	Mean	SD	Total	Weight	IV, Random, 95% CI
Arbillaga-Etxarri et al (2018)	0	10.8	132	-2	9.49	148	61.2%	2.00 [-0.39, 4.39]
Armstrong et al (2021)	11	9.4728	24	3	11.841	24	38.8%	8.00 [1.93, 14.07]
**Total (95% CI)**			**156**			**172**	**100.0%**	**4.33 [-1.40, 10.06]**

Heterogeneity: Tau² = 12.46; Chi² = 3.25, df = 1 (P = 0.07); I² = 69%
Test for overall effect: Z = 1.48 (P = 0.14)

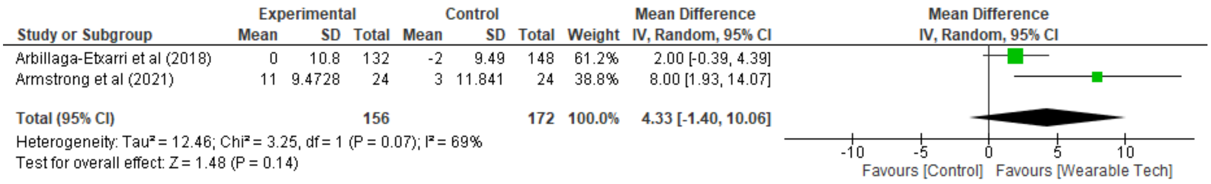

5b: Pooled results for the difficulty of exercise score

	Wearable Tech			Control				Mean Difference
Study or Subgroup	Mean	SD	Total	Mean	SD	Total	Weight	IV, Fixed, 95% CI
Arbillaga-Etxarri et al (2018)	3	9.49	132	-2	8.59	148	83.2%	5.00 [2.87, 7.13]
Armstrong et al (2021)	7	9.4728	24	-1	7.1046	24	16.8%	8.00 [3.26, 12.74]
**Total (95% CI)**			**156**			**172**	**100.0%**	**5.50 [3.56, 7.45]**

Heterogeneity: Chi² = 1.28, df = 1 (P = 0.26); I² = 22%
Test for overall effect: Z = 5.55 (P < 0.00001)

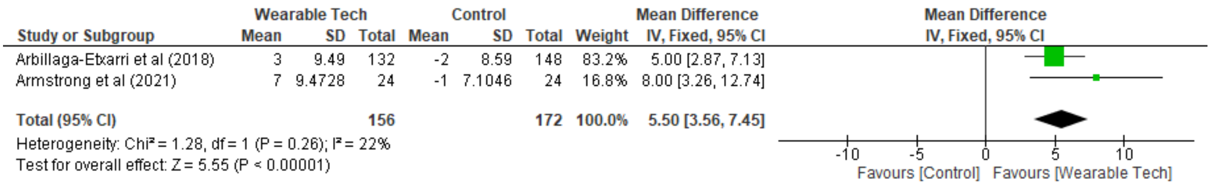

5c: Pooled results for total

	Wearable Tech			Control				Mean Difference
Study or Subgroup	Mean	SD	Total	Mean	SD	Total	Weight	IV, Random, 95% CI
Arbillaga-Etxarri et al (2018)	2	8.44	132	-2	7.59	148	56.6%	4.00 [2.11, 5.89]
Armstrong et al (2021)	9	7.1046	24	1	4.7364	24	43.4%	8.00 [4.58, 11.42]
**Total (95% CI)**			**156**			**172**	**100.0%**	**5.74 [1.85, 9.62]**

Heterogeneity: Tau² = 6.02; Chi² = 4.03, df = 1 (P = 0.04); I² = 75%
Test for overall effect: Z = 2.89 (P = 0.004)

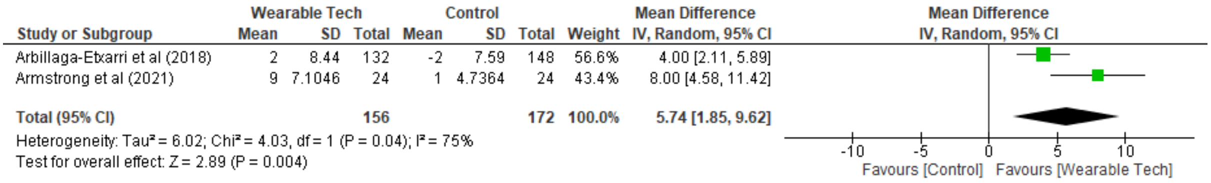

Supplementary Figure 6: Cochrane-risk-of-bias tool for randomised controlled trials

Study ID	D1	D2	D3	D4	D5	Overall
Alrajeh et al. 2020	+	!	+	+	+	!
Altenburg et al.2014	+	!	-	+	+	-
Arbillage-Etxarri et al. 2018	+	!	+	+	+	!
Armstrong et al (2021)	!	!	!	+	+	!
Bentley et al.2020	+	-	!	+	+	-
De Block et al. 2005	+	!	+	+	+	!
Demeyer et al. 2017	+	!	+	+	+	!
Geidl et al. 2022	+	+	+	+	+	+
Hornikx et al. 2015	+	!	+	+	+	!
Hospes et al. .2009	-	!	+	+	!	-
Kato et al. (2017)	+	-	+	+	+	-
Kawagoshi et al (2015)	!	!	!	+	+	!
Kohlbrenner et al (2020)	+	!	+	+	+	!
Mendoza et al. 2015	+	!	+	+	+	!
Nguyen et al.2009	+	+	+	+	+	+
Nguyen et al. 2019	+	+	+	+	+	+
Nolan et al. (2017)	+	!	+	+	+	!
Varas et al. (2018)	-	!	+	+	+	-
Vorrnik et al. 2016	+	-	+	+	+	-
Wan et all (2017)	+	!	+	+	+	!
Widyastuti et al(2018)	!	!	+	+	+	!
Wootton et al. 2017	+	!	+	+	+	!
Wootton et all (2019)	+	-	-	+	+	-

+	Low risk
!	Some concerns
-	High risk

D1	Randomisation process
D2	Deviations from the intended interventions
D3	Missing outcome data
D4	Measurement of the outcome
D5	Selection of the reported result

Benzo et al. 2021	-	!	+	+	+	-
Chen et al. 2022	!	-	!	+	+	-
Park et al. 2020	+	+	+	+	+	+
Robinson et al. 2021	+	+	+	+	+	+
Spielmanns et al. 2023	+	+	+	+	+	+
Valeiro et al. 2022	+	!	+	+	+	!

*Wan et al (2020) not included in this analysis as it was a secondary analysis to a previous RCT.

Supplementary Figure 7: Newcastle Ottawa Scale ratings for the observational studies

Author, Year	Population representative	Selection of non-exposed cohort	Exposure	Apriori Outcome	Comparability	Outcome assessment	Follow-up duration	Follow-up adequacy	Total rating (max 9)
Cooper et al (2019)	1	1	1	1	1	1	1	0	7
Hawthorne et al (2022)	1	1	1	0	1	1	1	1	7
Moy et al (2012)	1	1	1	1	2	1	0	1	8
Rubio et al (2017)	1	1	1	1	2	1	1	1	9
Sasaki et al (2022)	1	1	1	1	2	0	1	1	8
Wu et al (2021)	1	1	1	1	1	1	0	1	7

*Al Rajeh et al (2021) not included in this as it was a secondary analysis and the initial study has been included in the ROB assessment tool in Figure S8.

Supplementary Table 1: Multivariable meta-regression results for the mean daily step count

Covariate	Regression Coefficient	P-value	95% confidence interval
**Age**	-0.1498	0.40	-1.55 to 1.25
**Publication year**	0.2388	0.15	-0.99 to 1.47
**FEV1 (% predicted)**	-0.0465	0.40	-1.55 to 1.25
**Type of pedometer used for the intervention**			
**Fitbit Zip**	-0.2028	0.89	-14.63 to 14.23
**Fitburg**	0.4719	0.74	-13.09 to 14.03
**G-Sensor**	2.0192	0.31	-11.47 to 15.51
**Omron**	0.2123	0.80	-8.01 to 8.44
**PD724**	2.0231	0.37	-14.92 to 18.97
**Vivofit Activity Monitor**	-0.6227	0.73	-18.09 to 16.85
**Digi-walker**	2.2117	0.30	-12.23 to 16.65
**Outcome measurement device**			
**Dynaport accelerometer**	-0.3726	0.74	-11.36 to 10.61
**Omron pedometer**	0.2930	0.81	-11.83 to 12.42
**SenseWear Accelerometer**	-2.2625	0.29	-16.13 to 11.61
